# Toward the Total Synthesis of Sesquiterpene via an Annulative and Oxidative Approach

**DOI:** 10.1021/acsomega.5c03127

**Published:** 2025-07-25

**Authors:** Ajmir Khan, Fernando C. Rezende

**Affiliations:** † School of Packaging, Michigan State University, 448 Wilson Rd, East Lansing, Michigan 48824, United States; ‡ Department of Fundamental Chemistry, Institute of Chemistry, University of São Paulo, Av. Prof. Lineu Prestes, 748, São Paulo, SP 05508-000, Brazil

## Abstract

The phenolic sesquiterpene (±)-jungianol, originally isolated from *Jungia malvifolia* (family *Asteraceae*), has previously been targeted through various synthetic approaches. However, none of these methods have successfully produced (±)-jungianol as the major product, largely due to difficulties in synthesizing *trans*-1,3-substituted indane frameworks. Herein, we present an annulative strategy for the total synthesis of (±)-jungianol, emphasizing several key transformations, including olefination via Wittig and Grignard reactions, hydrogenation, iodination, cross-coupling reactions, oxidation, acetylation, and hydrogenolysis of the acetylated product. Most notably, this study explores the use of a ring contraction reaction, particularly for the formation of *trans*-1,3-substituted indanes, structural motifs essential to (±)-jungianol via an environmentally friendly iodine­(III) reagent. Efforts are underway to synthesize the targeted natural product, which is just one step away from our synthesized trans-indane intermediate.

## Graphical abstract

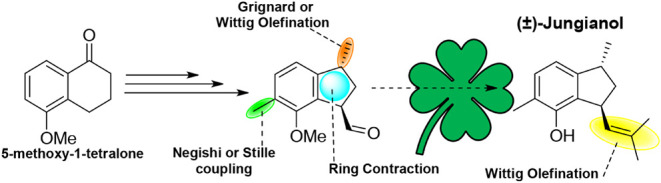

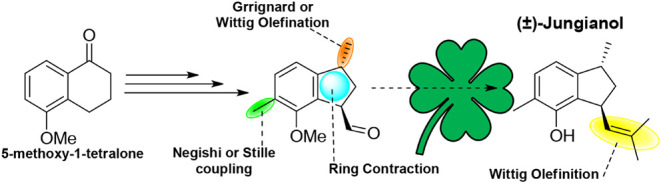

## Introduction

A phenolic sesquiterpene, (±)-jungianol (**1**) (in the racemic form), was isolated and characterized by Bohlmann et al. in 1977 from the roots of the South American plant *Jungia malvifolia* of the family *Asteraceae*.^1^ *Jungia malvifolia* (a synonym of *Jungia rugosa*) is traditionally known as “carne humana” in Ecuador and used in folk medicine for its anti-inflammatory and antioxidant properties.

(±)-Jungianol (**1**) features a tetrasubstituted indane framework with a methyl group at position 1 and an isobutenyl side chain at position 3 of the five-membered indane ring. Based on the ^1^H NMR spectra obtained after the isolation of (±)-jungianol (**1**), Bohlmann and coworkers initially reported that the compound exhibited a cis-configuration, with both substituents on the same face of the five-membered ring. However, the absolute configuration of the two stereogenic centers, as well as the optical rotation, remained unknown. Twenty years later, during a study involving the first total synthesis of (±)-mutisiantol (**15**), Ho et al. reviewed the structure of jungianol (**1**) and determined that the compound was in fact the trans-isomer, with the two substituents on opposite faces of the five-membered ring.^2^

Hashmi and coworkers investigated the first total synthesis of (±)-jungianol (**1**) using a gold-catalyzed intramolecular [4 + 2] cycloaddition between furan and an alkyne.^3^ The major product reported by Hashmi et al. was the unwanted cis-isomer *epi*-jungianol (**2**) in 68% yield, while the natural product (±)-jungianol (**1**) was isolated in only 21% yield (Scheme 1A). FeCl_3_-catalyzed Prins-type cyclization was used by Dethe and coworkers in 2013 to access the targeted natural product (±)-jungianol. However, the anticipated product was not isolated as the major product but with *epi*-jungianol in a 1:1 molar ratio (Scheme 1B).^4^ Later, in 2015, Dethe et al. again reported an alternative approach involving an FeCl_3_-catalyzed intramolecular Michael addition of a styrenic double bond onto an α,β-unsaturated carbonyl compound. This strategy resulted in the formation of a 1:1 molar mixture of (±)-jungianol (**1**) and its cis-isomer, *epi*-jungianol (**2**), as shown in Scheme 1C.^5,6^

**Scheme 1:**
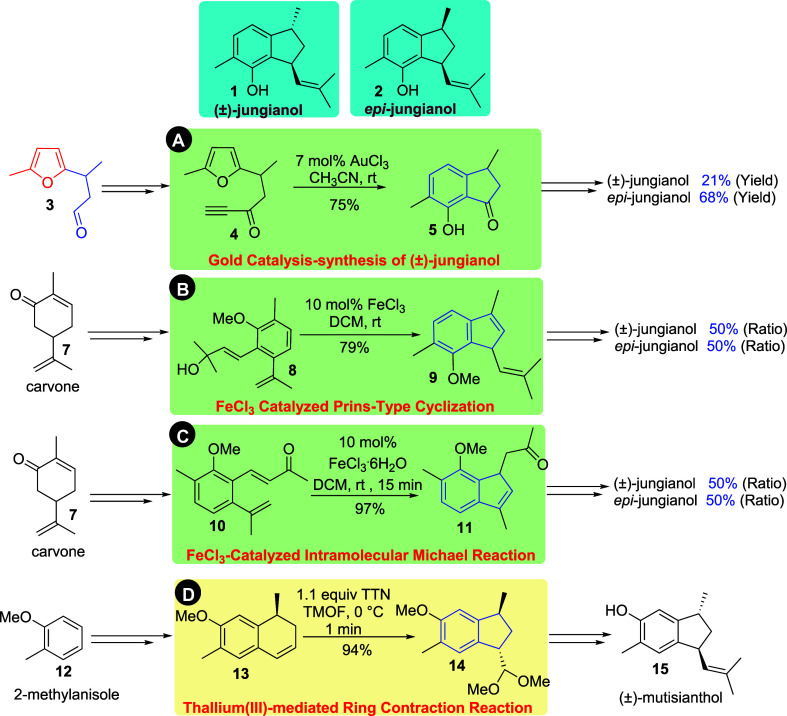
**A)** Representation of Hashmi Gold-Catalyzed Intramolecular [4 + 2] Cycloaddition; **B)** Dethe FeCl_3_-Catalyzed Prins-Type Cyclization; **C)** Dethe FeCl_3_-Catalyzed Intramolecular Michael Addition Reaction for the Total Synthesis of (±)-Jungianol; and **D)** Total Synthesis of (±)-Mutisiantol using a Key Step Ring Contraction Reaction

Previously, Silva and Bombonato also explored the ring contraction reaction in an attempt to synthesize (±)-jungianol, but they were unsuccessful in obtaining the target natural product.^7^ The strategies outlined in Scheme 1A–C thus highlight the efforts of various chemists who have developed efficient metal-catalyzed cyclization approaches toward the synthesis of (±)-jungianol (**1**); however, none of these methods have yielded (±)-jungianol as the major product. These studies underscore the challenges associated with accessing *trans*-1,3-substituted indanes.

In 2009, Silva et al. reported the first asymmetric total synthesis of a potent antitumor natural product (+)- and (−)-mutisiantol.^9^ Their strategy involved a key step ring contraction reaction of 1,2-dihydronaphthalene derivative **13** mediated by thallium­(III) nitrate (Tl­(NO_3_)_3_), performed in trifluoromethanol (TMOF) or methanol.^9^ This reaction efficiently provided *trans*-1,3-substituted indane **14** in 94% yield (Scheme 1D). This achievement not only enabled the assignment of the absolute configuration of mutisiantol but also provided valuable insights into the total synthesis of (±)-jungianol, which shares a similar indane framework.

Both thallium­(III) and iodine­(III) reagents are known to facilitate ring contraction reactions efficiently, particularly in the construction of *trans*-1,3-substituted indanes—structural motifs essential to (±)-jungianol and (±)-mutisiantol.^10^ However, while thallium­(III) is effective, it poses significant challenges due to its high toxicity and handling difficulties. In contrast, iodine­(III) reagents offer a more environmentally friendly and safer alternative, making them more suitable for sustainable synthetic applications.^11,12^

In this study, we set out to synthesize the natural product (±)-jungianol, starting from commercially available 5-methoxy-1-tetralone. Our strategy involved a total of nine linear steps, featuring a key step ring contraction reaction promoted by the environmentally friendly iodine­(III) reagent, HTIB (Hydroxy­(tosyloxy)­iodobenzene). The ring contraction of electron-rich 1,2-dihydronaphthalene derivatives proved to be a challenging step due to the high electron density of the aromatic system, which can complicate the formation of stable carbocation intermediates required for the ring contraction process during oxidative rearrangement reactions.^13^ However, by employing fluorinated alcohols instead of CH_3_CN, we achieved the desired ring contractions in good yields. The aim of this project was to accomplish the first synthesis of this molecule as a major product, as no such synthesis has been reported to date. Although the total synthesis of the target natural product has not yet been completed, our work has contributed valuable insights and robust methodologies for the construction of the indane skeleton—a core structure found in several natural products.

## Results and Discussion

Scheme 2 illustrates the retrosynthetic analysis aimed at synthesizing the natural product (±)-jungianol from the precursor 5-methoxy-1-tetralone that also highlights several key reaction steps.

**Scheme 2:**
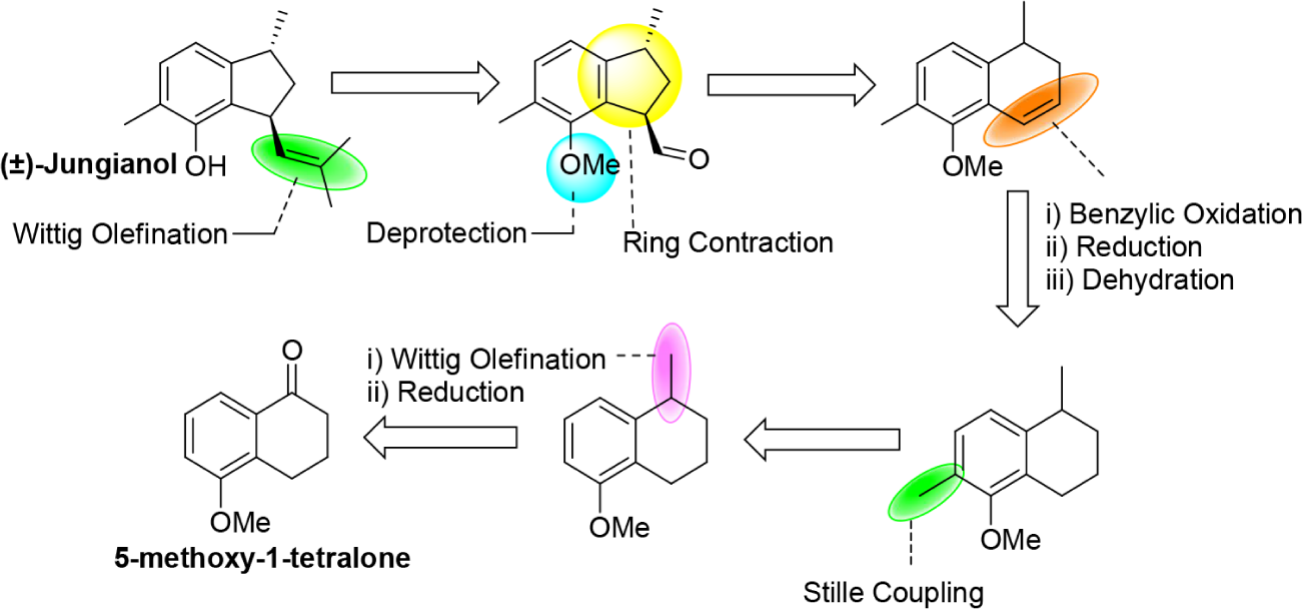
Retrosynthetic Analysis of *(*±)-Jungianol (**1**)

Starting from commercially available 5-methoxy-1-tetralone (**16**), alkenes **17** and **18** were obtained via Wittig olefination and a Grignard reaction, respectively.^14^ In the subsequent step, these alkenes were hydrogenated in methanol using a catalytic amount of Pd/C, affording desired product **19** in 99% yield (Scheme 3).

**Scheme 3:**
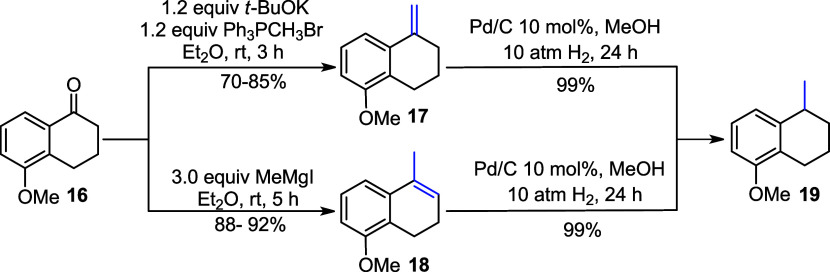
Olefination of **16** with Wittig and Grignard Reactions, Followed by Hydrogenation Reactions

Various reaction conditions were studied to convert compound **19** into the corresponding iodoarene **20**. In an initial attempt to abstract the ortho-hydrogen from the aromatic ring, *n*-BuLi was used as the base in the presence of tetramethylethylenediamine (TMEDA) to enhance the basicity of the *n*-butyl anion. 1,2-Diiodoethane served as the iodine source in *n*-hexane; however, no reaction occurred, and the starting material was recovered (entry 1, Table 1). Subsequently, a stronger base, *t*-BuLi, was employed with ICH_2_CH_2_I in *n*-hexane that successfully yielded iodoarene **20** in 70% (entry 2).^15^ On the other hand, when MeI was used as the electrophile under similar conditions, an unexpected hydroxylation at C-6 was observed. This may have occurred during the workup process, where washing with saturated aqueous NaHCO_3_ and brine could have promoted the formation of the hydroxylated product (entry 3). Further optimization involved using *t*-BuLi with iodine crystals (I_2_) in a solvent mixture of Et_2_O:DCM (4:1), which gave iodoarene **20** in 50% yield (entry 4). When the same reaction was conducted in diethyl ether alone, the yield improved to 66% (entry 5, Table 1).

**Table 1:**
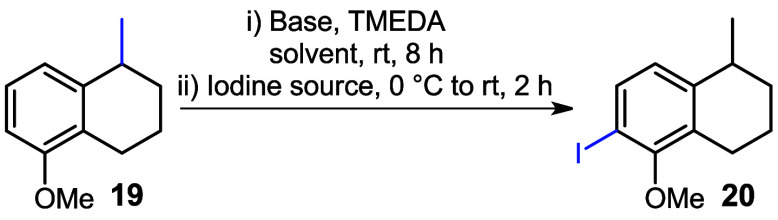

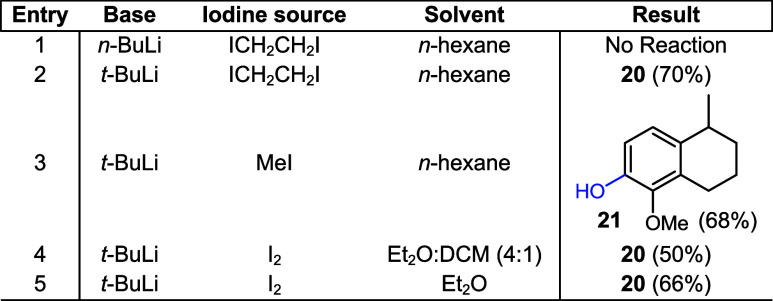
Optimization of the Formation of Iodoarene^a^

a In all cases, 0.25 equiv of tetramethylethylenediamine (TMEDA) were used.

Stille coupling reaction is widely used in synthetic organic chemistry especially in the total synthesis of natural products because of its functional group compatibility.^16^ Applying the literature procedure,^17^ iodoarene **20** was converted into **22** using tetramethyltin (Me_4_Sn) and a catalytic amount of bis­(triphenylphosphine)­palladium­(II) dichloride (Pd­(PPh_3_)_2_Cl_2_) in DMF (Scheme 4). The conversion of **20** to **22** was also brought by Negishi cross-coupling reaction, using dimethylzinc and a catalytic amount of Pd­(PPh_3_)_2_Cl_2_ in THF. To this reaction, triphenylphosphine (PPh_3_) was added with aims to activate the Pd catalyst that afforded the corresponding product **22** in 89% yield.

**Scheme 4:**
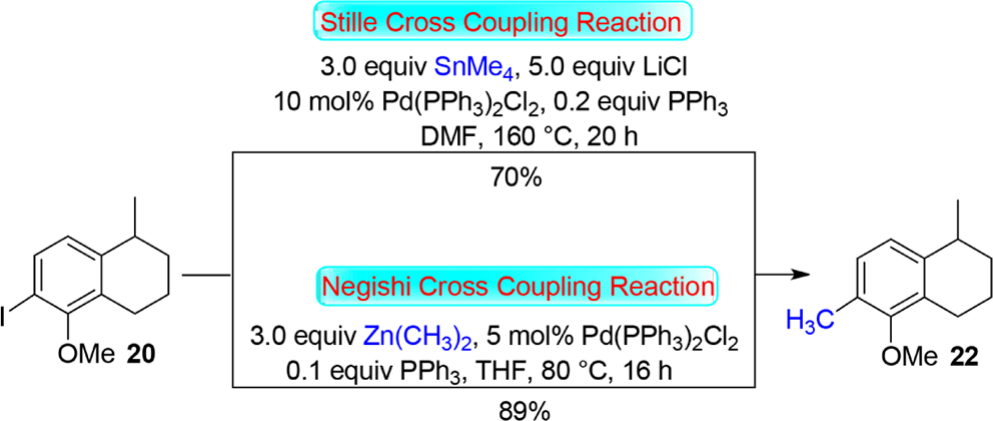
Stille and Negishi Cross-Coupling Reactions

The direct conversion of **19** to **22** was also carried out using *t*-BuLi and 3.0 equiv of MeI in hexane. The desired carbon–carbon coupling was successfully achieved, yielding compound **22** in 54% yield (Scheme 5).

**Scheme 5:**
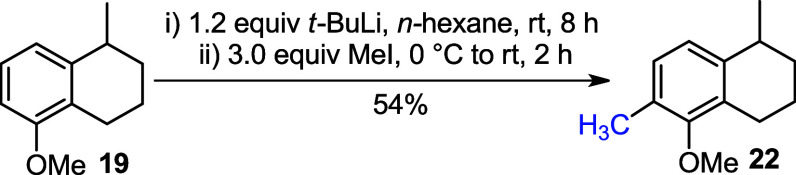
Formation of **22** via Ortho-Lithiation of **19**

Following a reported procedure of the litrature,^18^ KMnO_4_ was directly adsorbed onto the supporting surface such as MgSO_4_·7H_2_O in acetone. Substrate **22** was then reacted with the mixture of KMnO_4_–MgSO_4_·7H_2_O at 0 °C, affording compounds **23a**, **23b**, and **23c** in 42%, 27%, and 12% yields, respectively (Scheme 6). The singly oxidized product **23c** obtained from this reaction was further oxidized to **23a** in 92% yield, using the same procedure of reacting it with the mixture of KMnO_4_–MgSO_4_·7H_2_O at 0 °C (Scheme 6).

**Scheme 6:**
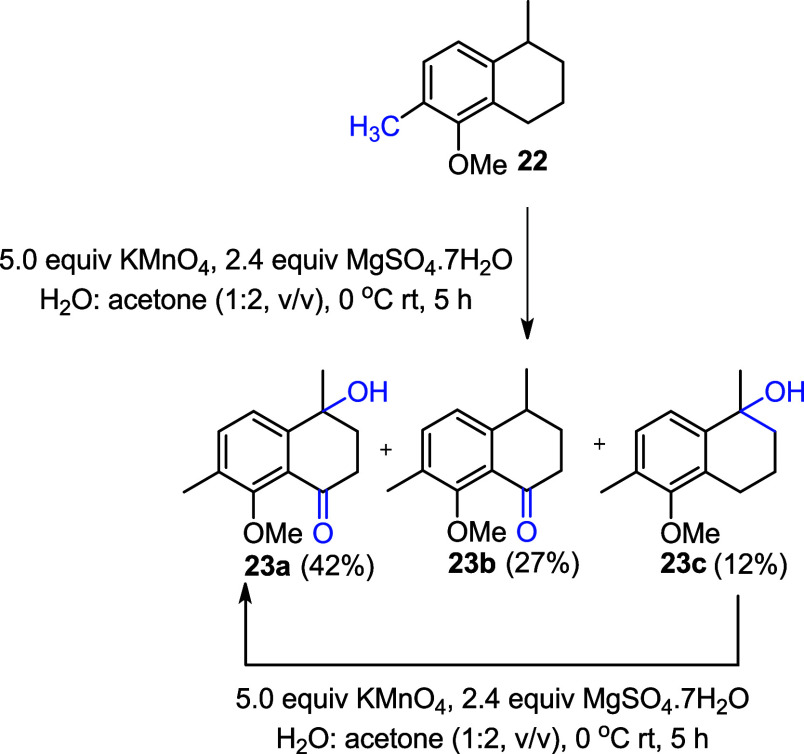
Benzylic Oxidation of **22**

Subsequently, ketone **23b** was subjected to a reduction reaction using NaBH_4_ to give alcohols (±)**25** in 95% yield (Scheme 7).

**Scheme 7:**
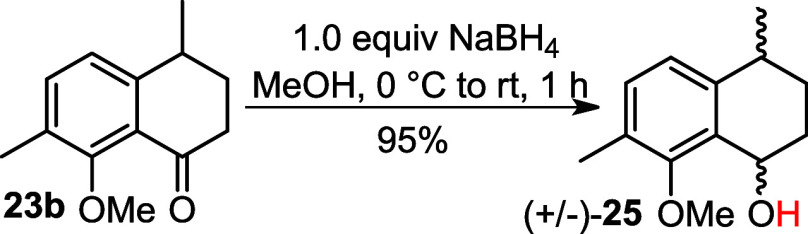
Reduction of Ketone into Alcohol (±)**25**

Alcohol **25** was identified by GC/MS, observing the molecular ion of *m*/*z* 206. Moreover, the absence of a carbonyl peak in ^13^C NMR has also confirmed the conversion of ketone **23b** into alcohol **25** (Figure 1).

**Figure 1:**
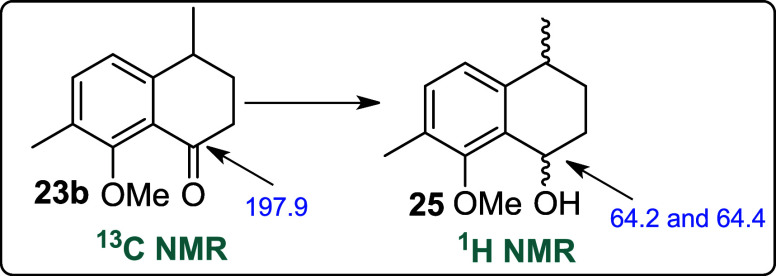
^13^C NMR data for ketone **23b** and alcohols (±)**25**.

As shown in Scheme 6, doubly oxidized compound **23a** was the major product when the oxidation was carried out with KMnO_4_. To remove the benzylic hydroxy group (−OH) present at position 1, acetylation was performed using 4-dimethylaminopyridine (DMAP) and acetic anhydride in EtOAc, following a reported literature procedure,^19^ affording the desired product **24** in 98% yield. Additionally, compound **23a** was also acetylated by using NaH as a base and acetyl chloride (AcCl) as the electrophile, affording the desired product **24** in 34% yield (Scheme 8).

**Scheme 8:**
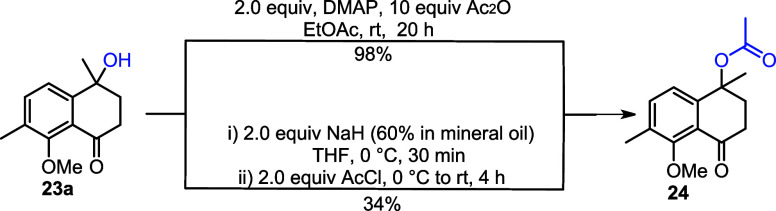
Preparation of Acetate **24** from Alcohol **23a**

Palladium on carbon (Pd/C) is one of the most valuable heterogeneous hydrogenation catalysts.^20^ Aromatic aldehydes and ketones are smoothly hydrogenolyzed to their corresponding methylene compounds via the formation of intermediate benzyl alcohols using a Pd/C catalyst. However, isolating these intermediates is extremely challenging due to the high reactivity and low selectivity of Pd/C. Sajiki and coworkers reported a heterogeneous palladium–ethylenediamine complex [Pd/C­(en)] for the chemoselective and mild hydrogenation of aromatic aldehydes, ketones, and *O*-benzyl protecting groups.^21^ The Pd/C­(en) catalyst was thus prepared in our lab by stirring a suspension of 10% Pd/C in the presence of ethylenediamine (en) in methanol (MeOH) as follows:

$$\mathrm{Pd}/C\overset{\underset{\mathrm{rt},48\;h}{\mathrm{Etylenediamine},\mathrm{MeOH}}}{\to }\mathrm{Pd}/C\left(\mathrm{en}\right)$$

Following a reported literature protocol,^22^ acetate **24** was dissolved in MeOH and subjected to a hydrogen atmosphere in the presence of 10 mol % Pd/C­(en) for 24 h, resulting in complete consumption of the starting material and formation of a mixture of ketone **23b** and alcohol **25**. In the subsequent step, the obtained filtrate was treated with NaBH_4_ in MeOH to reduce the ketone, affording alcohol **25** as a mixture of stereoisomers in an 81% yield (Scheme 9). Subsequently, alcohol **25** was subjected to a dehydration reaction using a catalytic amount of *p*-TsOH in THF under reflux at 100 °C for 2 h, yielding an unexpected aromatized product **26** in 91% yield. However, when the reaction temperature was lowered to 80 °C and the mixture of alcohol **25** and catalytic *p*-TsOH was refluxed for 12 h, the desired alkene **27** was obtained in 98% yield (Scheme 9).

**Scheme 9:**
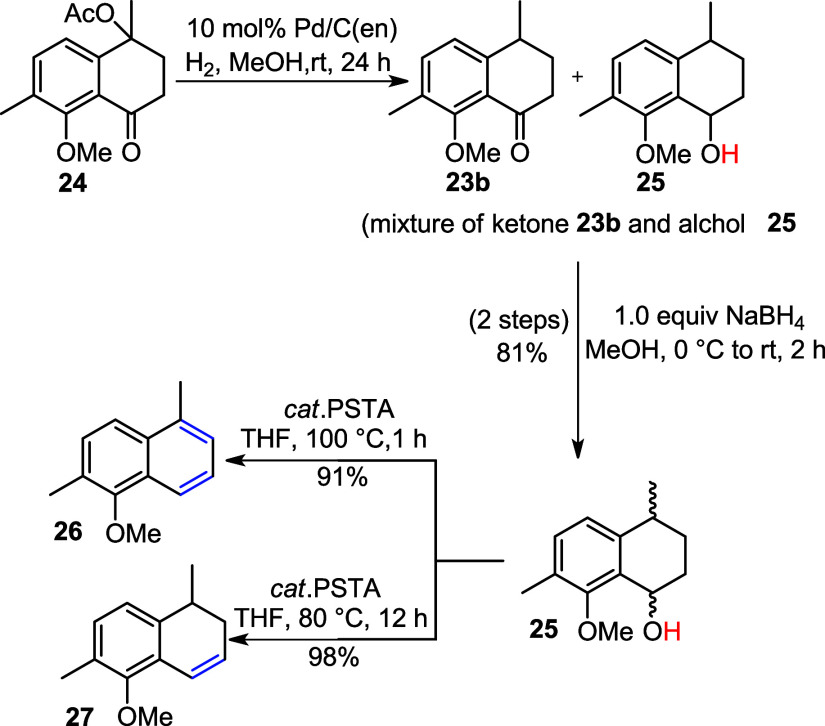
Hydrogenolysis of Acetylated Product **24** to the Corresponding Alcohol **25** Followed by its Aromatization **26** or Dehydration to Alkene **27**

HTIB (hydroxy­(tosyloxy)­iodobenzene), also known as Koser’s reagent, is a hypervalent iodine­(III) compound widely recognized as a versatile oxidant in organic synthesis. Based on previous studies that optimized ring contraction reactions for constructing indane frameworks, a variety of solvents were explored.^10,13,23–25^ In the key step of the total synthesis of (±)-jungianol, alkene **27** was first treated with HTIB in MeOH. However, TLC and GC-MS analyses revealed the formation of a complex mixture (entry 1, Table 2). Further attempts using HTIB in the fluorinated solvent HFIP (hexafluoroisopropanol) and in acetonitrile (CH_3_CN) at room temperature also resulted in complex product mixtures, as indicated by TLC and GC-MS (entries 2 and 3).

**Table 2:**
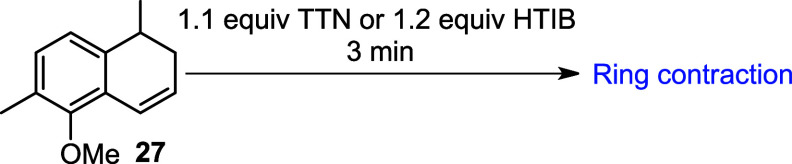

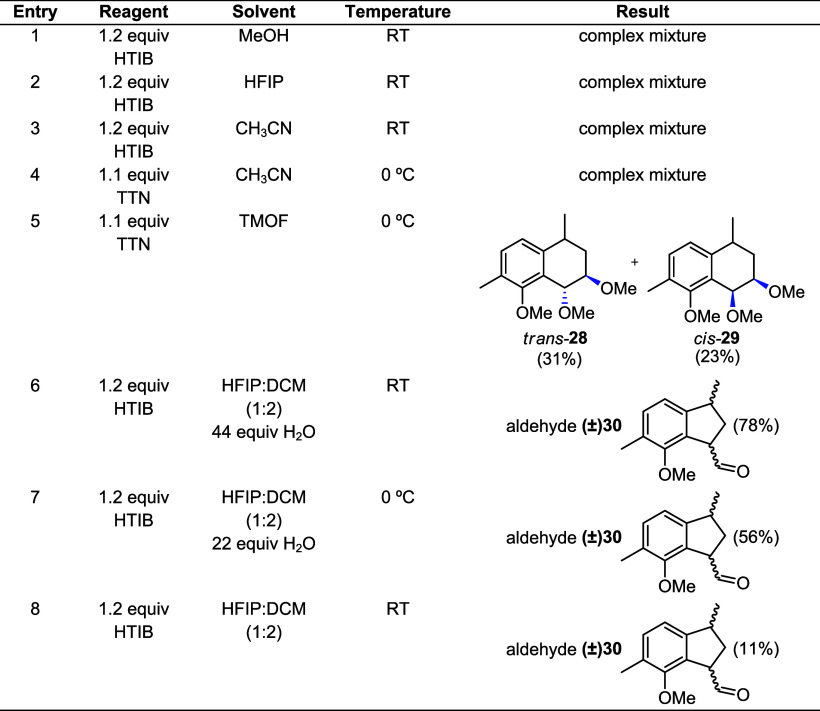
Oxidation of **27** using a Thallium­(III) Salt or Iodine­(III)^a^

a RT: room temperature; reaction time: 3 min.

An alternative oxidant, thallium salt or thallium­(III) nitrate trihydrate (Tl­(NO_3_)_3_·3H_2_O), was also reacted with alkene **27** for the possibility of to attempt the ring contraction reaction in CH_3_CN. The reaction progress was monitored by TLC after 3 min, and a mixture was observed (entry 4). However, when **27** was reacted with TTN in TMOF (trimethyl orthoformate) at 0 °C, trans*-* and cis-methoxylation products (**28** and **29**) were obtained in 31% and 23% yields, respectively (entry 5).

The trans*-* and cis-methoxylation products (**28** and **29**) were characterized by using NMR spectroscopy. According to the literature, benzylic carbons for cis-products show lower chemical shifts (78.0–78.3 ppm) compared to their trans-counterparts (78.8–80.1 ppm) in the corresponding ^13^C NMR spectra.^26^ Similarly, the benzylic hydrogen appears at lower chemical shift values for the cis-products than for their trans-counterparts in the ^1^H NMR spectra^26^ (see Supporting Information).

In subsequent reactions for ring contraction to obtain the five-membered ring product, alkene **27** was treated with HTIB in a solvent mixture of hexafluoroisopropanol (HFIP) and dichloromethane (DCM) in a 1:2 ratio, supplemented with 44 equiv of water, at room temperature. HFIP was used as the solvent due to its high polarity, low nucleophilicity, and high ionizing power to stabilize the cationic intermediates.^13^ On the other hand, DCM was added to improve substrate solubility, while water was used to enhance the solubility of HTIB. Under these conditions, the ring-contracted product, compound **30**, was obtained in a 78% yield (entry 6, Table 2). The structure of compound **30** was confirmed by ^1^H NMR spectroscopy, which indicated the presence of two diastereomers exhibiting both trans-configurations, as depicted in Figure 2.

**Figure 2:**
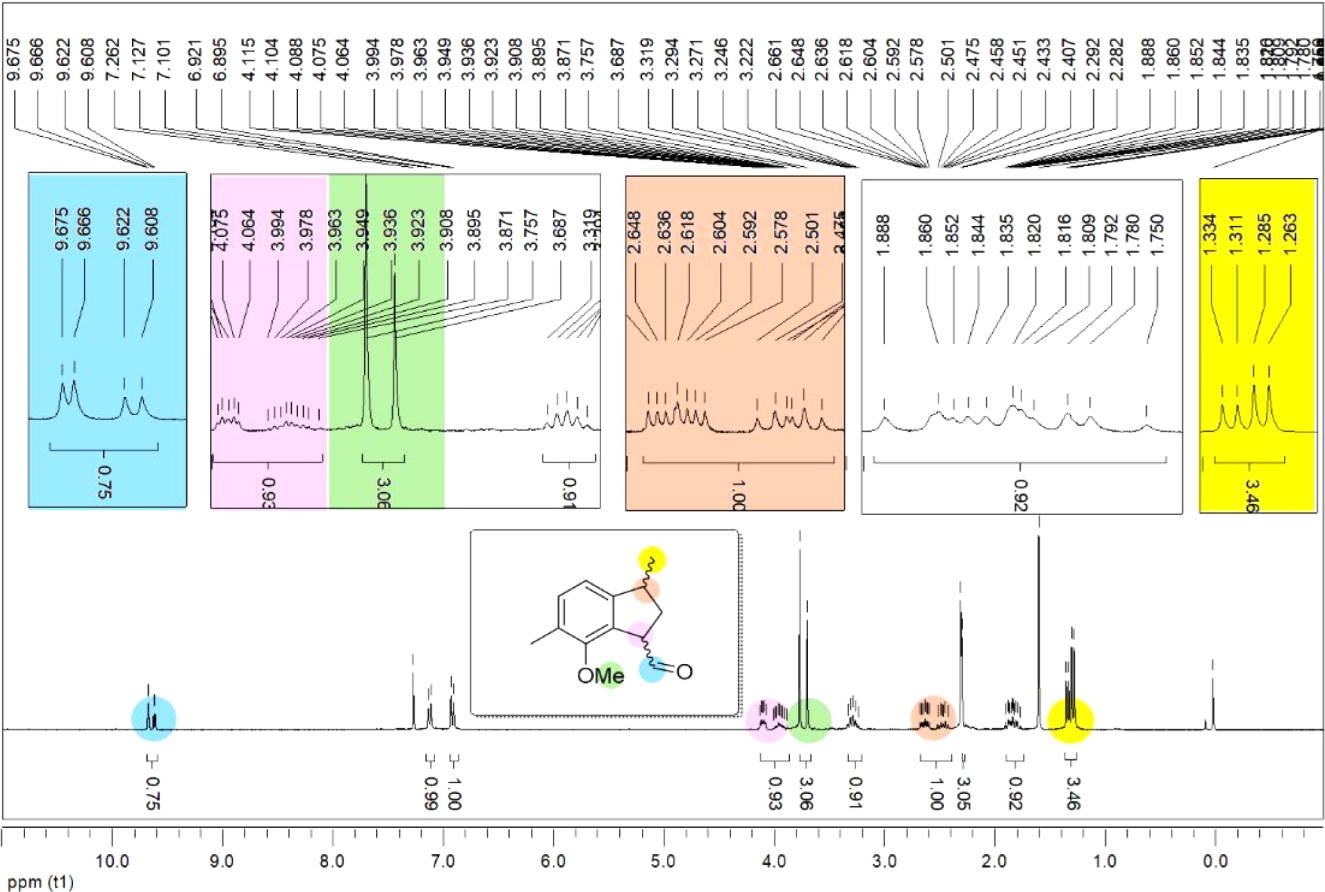
^1^H NMR spectroscopy indicates the presence of two diastereomers exhibiting both trans-configurations.

Notably, previous attempts at the total synthesis of jungianol by other researchers predominantly yielded the cis*-*isomer (*epi*-jungianol) as the major product (Scheme 1).^3–6^ In contrast, our approach, utilizing HTIB-mediated ring contraction, favored the formation of *trans*-1,3-disubstituted indane derivatives. This preference aligns with mechanistic insights from density functional theory (DFT) studies conducted by Braga and coworkers, which suggest that the stereoselectivity arises from the antiaddition of the iodine­(III) reagent to the alkene, leading to a benzylic carbocation intermediate that preferentially undergoes rearrangement to the trans-product due to minimized torsional strain and favorable antiperiplanar geometry.^27^

The reaction temperature was then lowered to 0 °C in an attempt to increase the yield of the corresponding indane, but the yield of product **30** dropped to 56% (entry 7). Next, the reaction was carried out using thallium salt (TTN) in HFIP:DCM (1:2), which yielded a mixture of indane in 11% yield (entry 8, Table 2).

The mechanism for the formation of trans-indane via the ring contraction reaction mediated by iodine (III) is shown in Scheme 10. The *anti* or *syn* addition of PhI­(+)­OH to the double bond of alkene **31** leads to the formation of benzylic carbocations **32A** or **32B**. As the ring contraction reaction in this study is conducted in a mixture of HFIP/DCM and water, we think that the use of fluorinated solvents such as HFIP with H_2_O, which possess high ionizing power compared to MeOH, facilitates the formation of PhI­(OH)^+^. According to Braga et al., the addition of PhIOH+ occurs preferentially in an *anti* orientation to the methyl group due to the less hindered side. In the case of *syn* addition, an eclipsing interaction occurs between the C–I bond and the β-allylic hydrogen, as well as the benzylic methyl group, which increases the torsional strain in **32B**.^27^ For the nucleophilic addition of the solvent, theoretical studies using the SMD continuum solvation method (Solvation Model based on Density) show that a cluster of three MeOH molecules adds to carbocation **32A**, rather than just a single molecule. These calculations also indicate that the trans-addition of the methanol cluster (MeOH)_3_ is energetically favored over the cis-addition, as the trans-addition results in lower free energy and potential energy.^12^

**Scheme 10:**
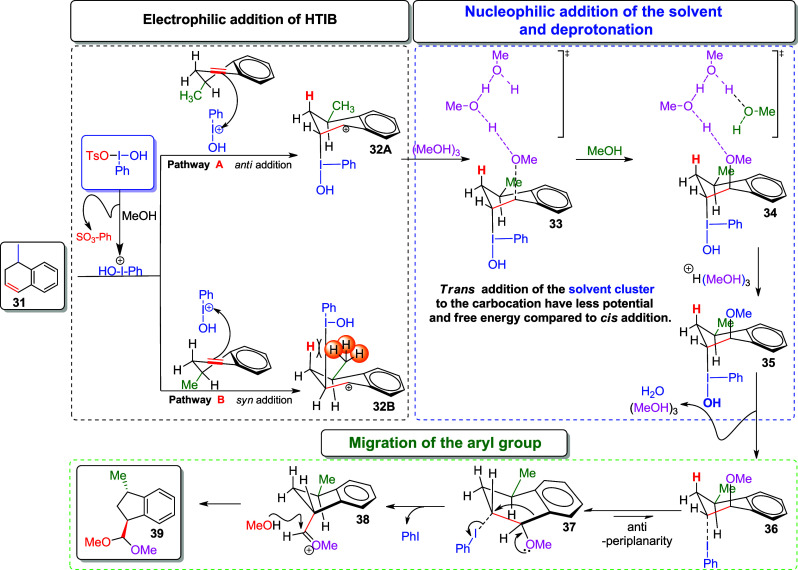
Ring Contraction Mechanism, Mediated by Iodine­(III)

In the final step, migration of the aryl group, along with protonation of the −OH group by the methanol cluster (+H­(MeOH)_3_), is followed by the reductive elimination of H_2_O to form intermediate **36**. After the elimination of H_2_O, the C–I bond weakens, facilitating the required antiperiplanar arrangement in **37**. In the subsequent step of the ring contraction, the formation of the C–C bond, followed by the loss of PhI, leads to the formation of trans-substituted five-membered ring **39**.

Due to the limited amount of available aldehyde (±)**30** (only 6 mg in hands), it was decided to convert it to the corresponding alkene (±)**40** via Wittig olefination, followed by deprotection, which would ultimately yield (±)-jungianol (±)**1** after demethylation. The aldehyde (±)**30** was then subjected to Wittig olefination in THF at 0 °C for 1 h, resulting in the formation of alkene (±)**40**. Next, the alkene (±)**40** was refluxed with NaSEt in DMF for 5 h to carry out the demethylation reaction (Scheme 11).^8^ However, TLC analysis of the final step revealed the formation of a complex mixture. GC–MS analysis of this mixture did not show any evidence of the targeted molecule, (±)**1**.

**Scheme 11:**
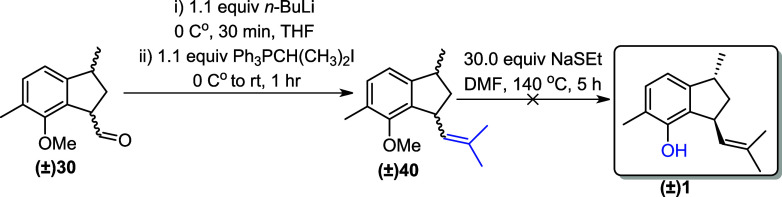
Wittig Olefination of Aldehyde **(±)­30**

Unfortunately, we were unable to isolate the alkenes (±)**40** by HPLC or any other technique due to the availability of a small amount (only 3 mg). The mixture of the two isomers was analyzed via NMR spectroscopy. However, the ^1^H and ^13^C NMR spectra were too complex and overlapped, preventing proper characterization of alkene (±)**40**. Nevertheless, high-resolution mass spectrometry (HRMS) confirmed the formation of alkenes (±)**40** by identifying their exact mass (Figure 3).

**Figure 3:**
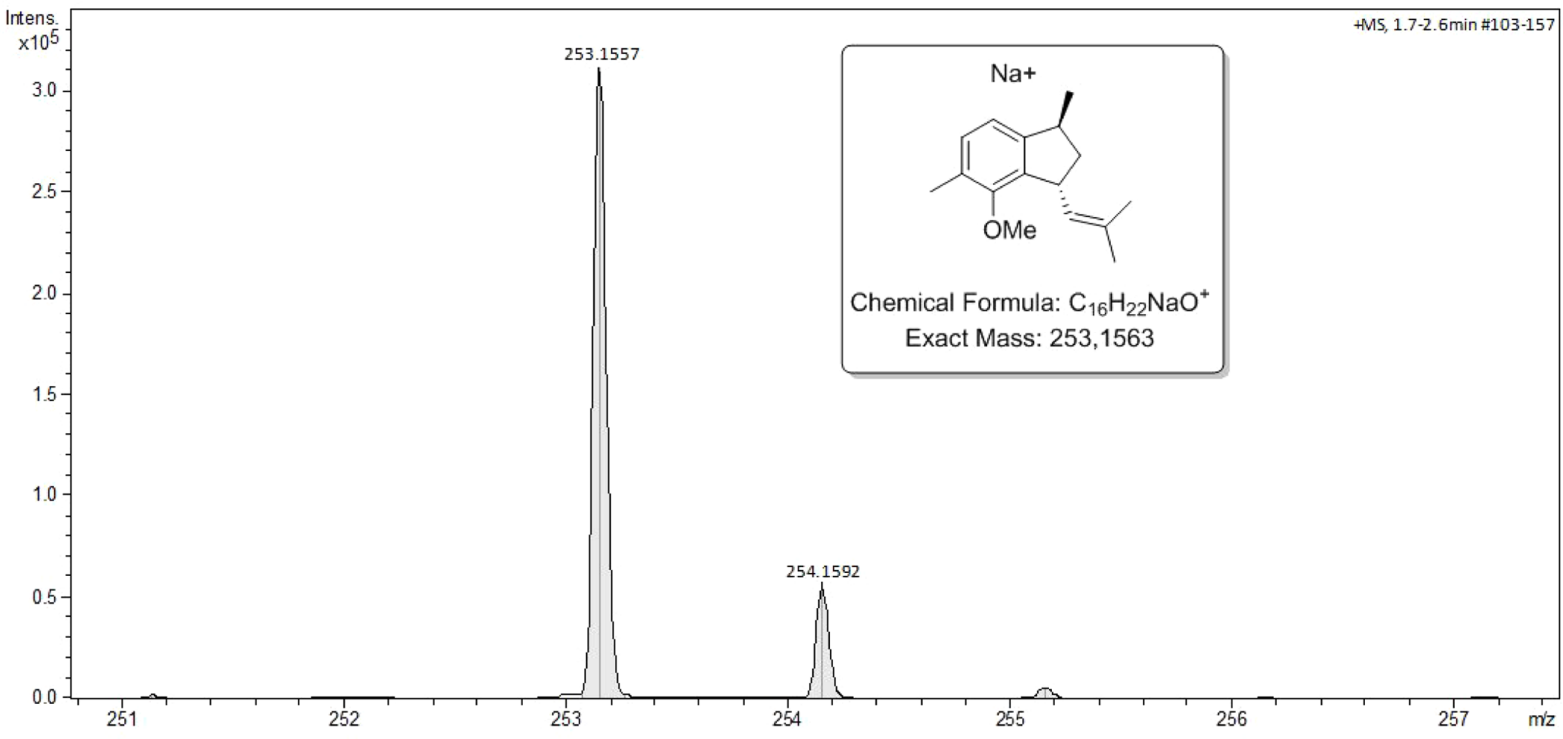
High-resolution mass spectrometry (HRMS) of alkene (±)**40**.

## Conclusion

To conclude, we described an approach toward the total synthesis of (±)-jungianol, starting from the commercially available 5-methoxy-1-tetralone. Our strategy involved several key steps, including olefination via Wittig and Grignard reactions, hydrogenation, iodination, cross-coupling reactions (Stille and Negishi), oxidation, acetylation, hydrogenolysis of the acetylated product, and most notably, a challenging ring contraction reaction of the electron-rich 1,2-dihydronaphthalene derivatives, promoted by an environmentally friendly iodine­(III) reagent. The aim of this project was to achieve the first reported total synthesis of this sesquiterpene ((±)-jungianol) as the major product. Although the complete total synthesis of the target natural product has not yet been accomplished due to the conclusion of the project, this work has nonetheless contributed valuable insights and robust methodologies for the construction of the indane skeleton—a core structure present in numerous natural products.

## Experimental Section

All reported compounds were characterized by ^13^C NMR and ^1^H NMR spectroscopy and compared with the literature data when available. New compounds were further characterized by ^1^H NMR, ^13^C NMR, IR, HRMS, and melting point (if solid). Wittig and Grignard reactions were performed in Schlenk flasks under a nitrogen atmosphere. Similarly, reduction reactions were carried out in septum-sealed flasks under nitrogen. Dehydration reactions were conducted in round-bottom flasks equipped with a Dean–Stark apparatus. Solvents and reagents were dried or treated as necessary, following standard procedures. Reaction progress was monitored by thin-layer chromatography (TLC) using Merck Type 60 F_254_ plates on aluminum, with visualization under UV light (254 nm) and staining with KMnO_4_ solution, phosphomolybdic acid solution, p-anisaldehyde, or vanillin. Most purifications were carried out using flash column chromatography (200–400 mesh silica gel). Chemical shifts for ^13^C and ^1^H NMR spectra are reported in parts per million (ppm), and coupling constants (*J*) are given in hertz (Hz). Signal multiplicities are abbreviated as follows: *s* = singlet; *d* = doublet; *dd* = doublet of doublets; *t* = triplet; *q* = quartet; *quin* = quintet; *br s* = broad singlet; *m* = multiplet. CDCl_3_ was used as the deuterated solvent with TMS as the internal reference (0 ppm).

### 1-Methoxy-5-methyl-5,6,7,8-tetrahydronaphthalen-2-ol (**21**)


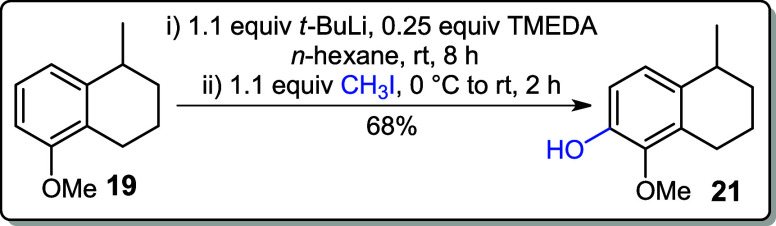


To a solution of **19** (0.176 g, 1.00 mmol) and tetramethylethylenediamine (TMEDA) (0.028 g, 0.25 mmol, 0.25 equiv), *t*-BuLi (0.91 M in pentane, 1.2 mL, 1.1 mmol, 0.70 g) was added dropwise in *n*-hexane (5 mL) at 0 °C. The resulting yellow, cloudy solution was stirred at room temperature for 8 h and then cooled back to 0 °C before the addition of methyl iodide (CH_3_I) (0.156 g, 1.10 mmol, 1.1 equiv). The reaction mixture was stirred for an additional 2 h at room temperature and then quenched with 1 M HCl (10 mL) followed by their extraction with EtOAc (3 × 10 mL). The combined organic layers were washed with saturated aqueous NaHCO_3_ and brine and dried over MgSO_4_, and the solvent was removed on a rotary evaporator. The concentrated crude product was purified by silica gel column chromatography (10% EtOAc in hexane).

**Yield:** 68% (0.130 g, 0.68 mmol).

**Sample appearance:** colorless oil.

^**1**^ **H NMR (300 MHz, CDCl**_**3**_ **) δ:** 1.25 (3H, d, *J* = 6.9 Hz), 1.44–1.53 (1H, m), 1.66–1.75 (1H, m), 1.80–1.93 (2H, m), 2.73 (2H, t, *J* = 5.2 Hz), 2.84 (1H, m), 3.77 (3H, s), 5.47 (1H, s), 6.78 (1H, d, *J* = 8.7 Hz), 6.89 (1H, d, *J* = 8.4 Hz).

^**13**^ **C NMR (75 MHz, CDCl**_**3**_ **) δ:** 20.0, 23.2, 24.1, 31.5, 32.1, 60.4, 113.0, 124.5, 130.5, 135.1, 144.3, 146.3.

**HRMS [ESI­(+)]** calcd. for [C_12_H_16_O_2_+Na]^+^ 215.1048, found 215.1031.

**IR (film):** 3413, 2932, 2866, 1863, 1607, 1490, 1454, 1430, 1373, 1296, 1241, 1081, 1037, 940, 861, 661 cm^–1^.

### 6-Iodo-5-methoxy-1-methyl-1,2,3,4-tetrahydronaphthalene (**20**)


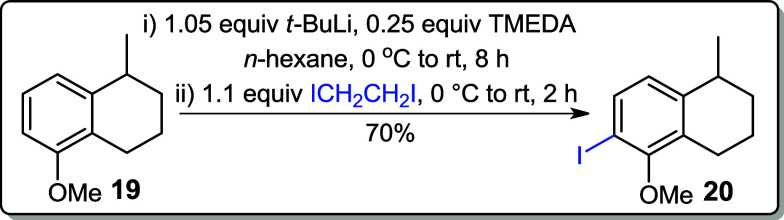


To the solution of **19** (0.352 g, 2.00 mmol) and TMEDA (0.058 g, 0.52 mmol, 0.25 equiv) in *n*-hexane (10 mL) at 0 °C was added *t*-BuLi (0.91 M in pentane, 0.134 g, 2.1 mmol, 2.3 mL) dropwise. The resulting yellow cloudy solution was stirred at rt for 8 h and then cooled to 0 °C for the addition of 1,2-diiodoethane (0.620 g, 2.20 mmol, 1.1 equiv). The resulting slurry was allowed to warm to rt and stirred for the next 2 h. The reaction mixture was diluted with hexane (20 mL) and HCl (1 M, 10 mL). The organic layer was extracted with EtOAc (3 × 10 mL), washed with aqueous NaHCO_3_ (10 mL) and brine (10 mL), and concentrated under reduced pressure. The crude product was purified by silica gel chromatography (in hexane).

**Yield:** 70% (0.422 g, 1.40 mmol).

**Sample appearance:** colorless oil.

^**1**^ **H NMR (300 MHz, CDCl**_**3**_ **) δ:** 1.26 (3H, d, *J* = 6.9 Hz), 1.47–1.57 (1H, m), 1.64–1.78 (1H, m), 1.80–1.93 (2H, m), 2.73–2.80 (2H, m), 2.83–2.92 (1H, m), 3.76 (3H, s), 6.74 (1H, d, *J* = 8.4 Hz), 7.53 (1H, d, *J* = 8.4 Hz).

^**13**^ **C NMR (75 MHz, CDCl**_**3**_ **) δ:** 19.9, 23.0, 24.8, 30.9, 32.4, 60.0, 87.9, 126.2, 132.2, 136.0, 144.9, 157.3.

**HRMS [ESI­(+)]** calcd. for [C_12_H_15_IO+Na]^+^ 325.0060, found 325.0059.

**IR (film):** 3060, 2932, 2866, 1560, 1460, 1398, 1321, 1231, 1077, 1062, 1006, 808 cm^–1^.

### 5-Methoxy-1,6-dimethyl-1,2,3,4-tetrahydronaphthalene (**22**)


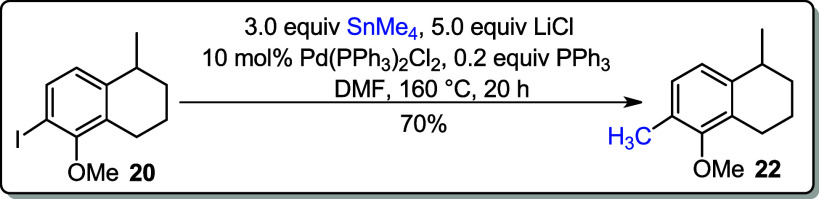


To the solution of **20** (0.269 g, 0.89 mmol) in DMF (6 mL) was added LiCl (0.189 mg, 4.45 mmol, 5.0 equiv), SnMe_4_ (0.477 mL, 2.67 mmol, 3.0 equiv), Pd­(PPh_3_)_2_Cl_2_ (10 mol %, 0.063 mg, 0.09 mmol), and PPh_3_ (0.048 mg, 0.18 mmol). The reaction flask was fitted with a condenser, purged with nitrogen, and refluxed for 20 h at 160 °C. After complete consumption of the starting materials, analyzed by TLC, the reaction mixture was allowed to cool, filtered through a short pad of Celite, and rinsed with EtOAc (20 mL). The organic layer was washed with H_2_O (3 × 15 mL) and brine (1× 15 mL). The organic fraction was dried over anhydrous MgSO_4_ and concentrated under reduced pressure. The residue was purified by silica gel chromatography (in hexane).

**Yield:** 70% (0.119 g, 0.63 mmol).

**Sample appearance:** colorless oil.

^**1**^ **H NMR (300 MHz, CDCl**_**3**_ **) δ:** 1.26 (3H, d, *J* = 6.9 Hz), 1.45–1.54 (1H, m), 1.62–1.75 (1H, m), 1.78–1.93 (2H, m), 2.25 (3H, s), 2.70–2.77 (2H, m), 2.81–2.96 (1H, m), 3.70 (3H, s), 5.47 (1H, s), 6.90 (1H, d, *J* = 7.8 Hz), 6.97 (1H, d, *J* = 7.8 Hz).

^**13**^ **C NMR (75 MHz, CDCl**_**3**_ **) δ:** 16.0, 20.2, 23.1, 24.0, 31.3, 32.5, 59.5, 123.8, 127.5, 128.2, 130.4, 141.7, 156.2.

### 5-Methoxy-1,6-dimethyl-1,2,3,4-tetrahydronaphthalene (**22**)


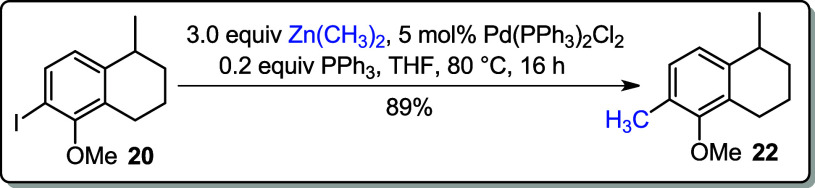


To a dry two-necked flask fitted with a condenser were added Pd­(PPh_3_)_2_Cl_2_ (5 mol %, 0.350 g, 0.500 mmol, 0.5 equiv) and PPh_3_ (0.052 g, 0.200 mmol, 0.2 equiv). The system was evacuated and purged three times with nitrogen. Next, the solution of iodoarene **20** (0.302 g, 1.00 mmol) in anhydrous THF (15 mL) was added to the flask. To the above mixture was added (CH_3_)_2_Zn (1.5 M in toluene, 0.286 g, 3.00 mmol, 2.0 mL) dropwise at rt. The reaction mixture was refluxed and allowed to stir for 16 h. After this period, the reaction showed an intense dark stain and was cooled to 0 °C and poured into 10% cold HCl solution. The organic layer was washed with H_2_O (3 × 10 mL) and brine (1 × 10 mL). The organic fraction was dried over anhydrous MgSO_4_ and concentrated under reduced pressure. The crude product was purified by silica gel chromatography (2% DCM in hexane).

**Yield:** 89% (0.169 g, 0.89 mmol).

^**1**^ **H NMR (300 MHz, CDCl**_**3**_ **) δ:** data given above.

^**13**^ **C NMR (75 MHz, CDCl**_**3**_ **) δ:** data given above.

### 5-Methoxy-1,6-dimethyl-1,2,3,4-tetrahydronaphthalene (**22**)


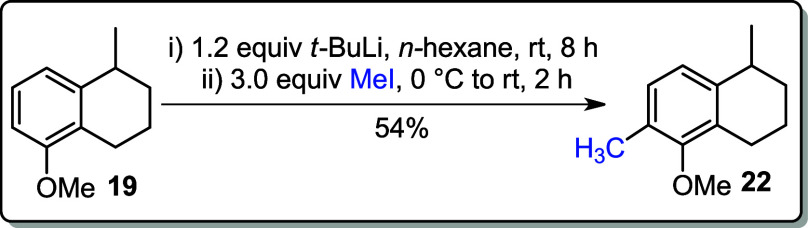


To the solution of **19** (0.352 g, 2.00 mmol) in hexane (10 mL) at 0 °C was dropwise added *t*-BuLi (0.91 M in pentane, 0.154 g, 2.40 mmol, 2.64 mL). The resulting yellow cloudy solution was stirred at rt for 8 h and then cooled to 0 °C for the addition of CH_3_I (0.852 g, 6.00 mmol, 3.0 equiv). The resulting mixture was allowed to warm to rt and was stirred for 2 h. The reaction mixture was diluted with hexane (20 mL) and HCl (1 M, 15 mL). The organic layer was extracted with EtOAc (3 × 15 mL), washed with aqueous NaHCO_3_ (20 mL) and brine (20 mL), and concentrated under reduced pressure. The crude product was purified by silica gel chromatography (in hexane).

**Yield:** 54% (0.205 g, 1.08 mmol).

^**1**^ **H NMR (300 MHz, CDCl**_**3**_ **) δ:** data given above.

^**13**^ **C NMR (75 MHz, CDCl**_**3**_ **) δ:** data given above.

### 4-Hydroxy-8-methoxy-4,7-dimethyl-3,4-dihydronaphthalen-1­(2*H*)-one (**23a**), 8-methoxy-4,7-dimethyl-3,4-dihydronaphthalen-1­(2*H*)-one (**23b**), and 5-methoxy-1,6-dimethyl-1,2,3,4-tetrahydronaphthalen-1-ol (**23c**)


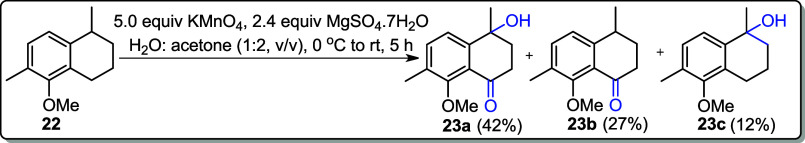


To a stirred solution of **22** (0.760 g, 4.00 mmol) in acetone (20 mL) was added MgSO_4_·7H_2_O (2.366 g, 9.60 mmol, 2.4 equiv) and H_2_O (10 mL) at 0 °C. To this mixture KMnO_4_ (3.160 g, 20.00 mmol, 5.0 equiv) was added in small portions over 30–40 min at 0 °C. Next, the reaction mixture was allowed to reach rt and stirred for 5 h. The solid was filtered, and the filtrate was treated with a saturated solution of K_2_S_2_O_5_ (20 mL). The resulting mixture was again filtered, and the filtrate was extracted with DCM (3 × 20 mL). The combined extract was washed with distilled water (25 mL) and saturated brine (25 mL) and dried over anhydrous MgSO_4_. The solvent was removed under reduced pressure. The crude was purified by silica gel chromatography (10–50% EtOAc in hexane).

#### 4-Hydroxy-8-methoxy-4,7-dimethyl-3,4-dihydronaphthalen-1­(2*H*)-one (**23a**)

**Yield:** 42% (0.370 g, 1.68 mmol).

**Sample appearance:** colorless oil.

^**1**^ **H NMR (300 MHz, CDCl**_**3**_ **) δ:** 1.57 (3H, s), 2.14–2.26 (5H, m), 2.56–2.67 (1H, m), 2.75–2.83 (1H, m), 3.75 (3H, s), 7.36 (2H, s).

^**13**^ **C NMR (75 MHz, CDCl**_**3**_ **) δ:** 15.8, 29.4, 37.2, 37.6, 61.0, 70.2, 120.5, 124.1, 132.1, 136.3, 149.6, 158.1, 197.0.

**HRMS [ESI­(+)]** calcd. for [C_13_H_16_O_3_+Na]^+^ 243.0992, found 243.0982.

**IR (film):** 3505, 3334, 3065, 2932, 1741, 1725, 1666, 1521, 1489, 1440, 1391, 1372, 1310, 1217, 1124, 1085, 988, 971, 885, 840, 799, 780, 736 cm^–1^.

#### 8-Methoxy-4,7-dimethyl-3,4-dihydronaphthalen-1­(2*H*)-one (**23b**)

**Yield:** 27% (0.220 g, 1.08 mmol).

**Sample appearance:** yellowish oil.

^**1**^ **H NMR (300 MHz, CDCl**_**3**_ **) δ:** 1.35 (3H, d, *J =* 6.9 Hz), 1.78–1.89 (1H, m), 2.10–2.24 (1H, m), 2.27 (3H, s), 2.52–2.64 (1H, m), 2.71–2.82 (1H, m), 2.95–3.05 (1H, m), 3.79 (3H, s), 6.98 (1H, d, *J =* 7.8 Hz), 7.31 (1H, d, *J =* 7.8 Hz).

^**13**^ **C NMR (75 MHz, CDCl**_**3**_ **) δ:** 15.8, 21.2, 30.1, 33.5, 37.9, 61.1, 122.8, 125.6, 130.9, 135.9, 149.0, 158.8, 197.9.

**HRMS [ESI­(+)]** calcd. for [C_13_H_16_O_2_+Na]^+^ 227.1048, found 227.1041.

**IR (film):** 3330, 3109, 3055, 2935, 1747, 1755, 1669, 1666, 1523, 1444, 1395, 1375, 1334, 1308, 1208, 1134, 1085, 999, 991, 971, 887, 795, 788, 718 cm^–1^.

#### 5-Methoxy-1,6-dimethyl-1,2,3,4-tetrahydronaphthalen-1-ol (**23c**)

**Yield:** 12% (0.099 g, 0.48 mmol).

**Sample appearance:** light red oil.

^**1**^ **H NMR (300 MHz, CDCl**_**3**_ **) δ:** 1.54 (3H, s), 1.73–1.94 (6H, m), 2.26 (3H, s), 2.73–2.79 (1H, m), 3.70 (3H, s), 7.05 (1H, d, *J =* 7.8 Hz), 7.29 (1H, d, *J =* 8.1 Hz).

^**13**^ **C NMR (75 MHz, CDCl**_**3**_ **) δ:** 16.0, 20.0, 24.0, 31.0, 39.6, 59.5, 70.7, 121.9, 129.0, 129.4, 130.0, 142.3, 155.8.

**HRMS [ESI­(+)]** calcd. for [C_13_H_18_O_2_+Na]^+^ 229.1204, found 229.1195.

**IR (film):** 3566, 3443, 3430, 3323, 3112, 3050, 2940, 1740, 1735, 1652, 1633, 1454, 1363, 1334, 1318, 1223, 1134, 1089, 1017, 999, 912, 888, 784, 773, 734 cm^–1^.

### 5-Methoxy-1,6-dimethyl-4-oxo-1,2,3,4-tetrahydronaphthalen-1-yl acetate (**24**)


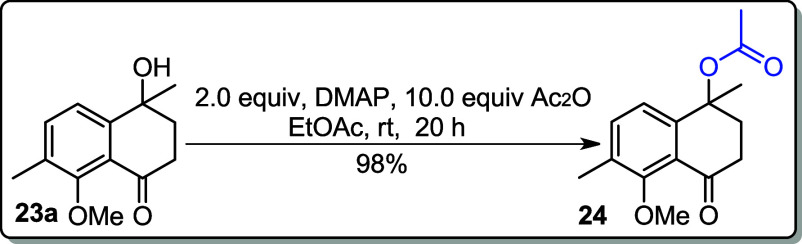


To the solution of **23a** (0.550 mg, 2.50 mmol) and DMAP (0.610 mg, 5.00 mmol) in EtOAc (10 mL), Ac_2_O (1.021 g, 10.00 mmol, 10.0 equiv) was added under a nitrogen atmosphere. The reaction mixture was stirred at room temperature for 20 h. After the completion of the reaction, the mixture was washed with H_2_O (20 mL), and the organic phase was dried over MgSO_4_. The solvent was removed under reduced pressure. The crude residue was purified by flash chromatography on silica gel (33–40% EtOAc in hexane).

**Yield:** 98% (0.677 g, 2.45 mmol).

**Sample appearance:** colorless oil.

^**1**^ **H NMR (300 MHz, CDCl**_**3**_ **) δ:** 1.86 (3H, s), 1.99 (3H, s), 2.24–2.32 (4H, m), 2.59–2.83 (3H, m), 3.80 (3H, s), 7.24 (1H, d, *J =* 8.1 Hz), 7.38 (1H, d, *J =* 8.1 Hz).

^**13**^ **C NMR (75 MHz, CDCl**_**3**_ **) δ:** 15.7, 22.0, 26.0, 33.2, 36.2, 61.2, 79.3, 120.9, 124.7, 132.6, 135.6, 145.0, 158.0, 169.7, 196.3.

**HRMS [ESI­(+)]** calcd. for [C_15_H_14_O_4_+H]^+^ 263.1278, found 263.1290.

**IR (film):** 3329, 2940, 1740, 1735, 1677, 1655, 1639, 1459, 1370, 1363, 1334, 1315, 1275, 1223, 1119, 1089, 1020, 1003, 988, 919, 881, 783, 777 cm^–1^.

### 5-Methoxy-1,6-dimethyl-4-oxo-1,2,3,4-tetrahydronaphthalen-1-yl acetate (**24**)


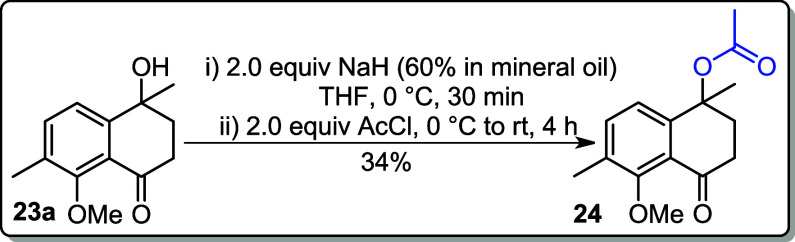


To a round-bottom flask, **23a** (0.220 g, 1.00 mmol) and NaH (0.080 g, 2.00 mmol, 60% dispersed in mineral oil) were added in THF (5 mL). After 30 min, AcCl (0.157 g 2.00 mmol) was added to the above solution at 0 °C. The reaction mixture was allowed to reach rt and stirred for 4 h. The reaction was quenched with distilled H_2_O (10 mL) and extracted with EtOAc (3 × 10 mL). The combined organic layers were washed with brine (10 mL), dried over MgSO_4_, and filtered. The solvent was removed under reduced pressure. The crude product was purified by flash column chromatography (33–40% EtOAc in hexane).

**Sample appearance:** colorless oil.

**Yield:** 34% (0.089 g, 0.34 mmol).

^**1**^ **H NMR (300 MHz, CDCl**_**3**_ **) δ:** data given above.

^**13**^ **C NMR (75 MHz, CDCl**_**3**_ **) δ:** data given above.

### The Mixture of 8-Methoxy-4,7-dimethyl-3,4-dihydronaphthalen-1­(2*H*)-one (**23b**) and 8-methoxy-4,7-dimethyl-1,2,3,4-tetrahydronaphthalen-1-ol (**25**)


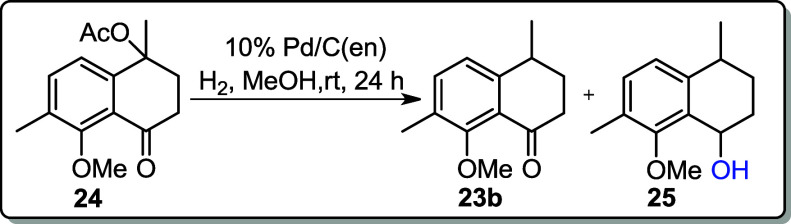


An autoclave was charged with **24** (0.524 g, 2.00 mmol) dissolved in anhydrous MeOH (10 mL) and 10% (w/w) Pd/C­(en) (0.053 g). The reaction mixture was purged 3 times with H_2_, and the autoclave was hydrogenated with 2 atm of H_2_. The reaction mixture described above was stirred for 24 h at rt. After completion of the reaction, it was filtered through a silica gel pad (ca. 10 cm) using DCM as the eluent to remove the coal and catalyst. The filtrate was concentrated in vacuo, giving a crude mixture of **23a** and **25** (0.405 g) as light-yellow oil and was proceeded to the next step without purification.

### 8-Methoxy-4,7-dimethyl-1,2,3,4-tetrahydronaphthalen-1-ol (**25**)


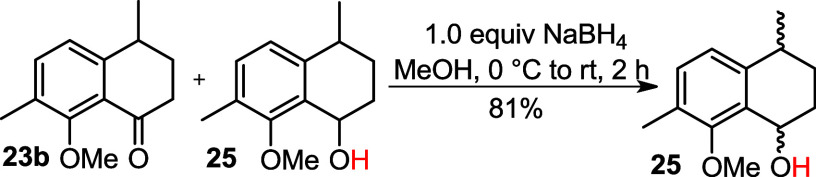


To a round-bottom flask, the mixture of ketone **23a** and alcohol **25** (0.405 g) dissolved in anhydrous MeOH (6 mL) and NaBH_4_ (0.076 g, 2.00 mmol) was added under N_2_ atmosphere at 0 °C. The mixture was warmed to rt and stirred for 2 h. After complete consumption of ketone **23b** (TLC analysis), the reaction was quenched by addition of distilled H_2_O (10 mL), and the mixture was extracted with EtOAc (3 × 10 mL). The combined organic extracts were washed with brine (10 mL) and dried in anhydrous MgSO_4_. The solvent was removed under pressure, and the crude residue was purified by flash chromatography on silica gel (20% EtOAc in hexane).

**Yield:** 81% (0.334 g, 1.62 mmol).

**Sample appearance:** light-yellow oil.

^**1**^ **H NMR (300 MHz, CDCl**_**3**_ **) δ:** spectrum is the mixture of two diastereomers (see Figure S21).

^**13**^ **C NMR (75 MHz, CDCl**_**3**_ **) δ:** spectrum is the mixture of two diastereomers (see Figure S22).

**HRMS [ESI­(+)]** calcd. for [C_13_H_19_O_2_+H]^+^ 207.1385, found 207.1380.

**LRMS** *m*/*z* (%): 206 (M^**+•**^, 64), 188 (82), 173 (100), 158 (74), 149 (62), 128 (46), 115 (68), 91 (60), 77 (39).

**IR (film):** 3531, 3335, 3131, 2929, 1735, 1677, 1658, 1620, 1541, 1459, 1421, 1370, 1371, 1339, 1330, 1215, 1153, 1099, 1025, 991, 881, 877, 744 cm^–1^.

### 1-Methoxy-2,5-dimethylnaphthalene (**26**)


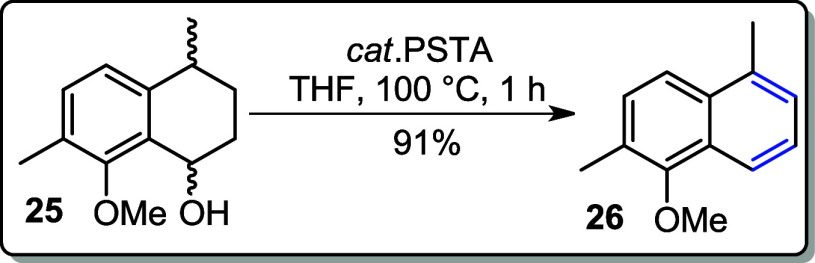


To a round-bottom flask fitted with a reflux condenser, alcohol **25** (0.206 g, 1.00 mmol) in THF (6 mL) was added with a catalytic amount of PTSA (*p*-TsOH.H_2_O) and refluxed between 100 and 105 °C for 1 h. The reaction was allowed to cool, quenched with saturated solution of NaHCO_3_, and extracted with EtOAc (3 × 10 mL). The combined organic layers were washed with brine (10 mL) and dried over anhydrous MgSO_4_. The solvent was removed at reduced pressure, and the crude was purified by flash chromatography (hexane).

**Yield:** 91% (0.170 g, 0.91 mmol).

**Sample appearance:** colorless oil.

^**1**^ **H NMR (300 MHz, CDCl**_**3**_ **) δ:** 2.46 (3H, s), 2.66 (3H, s), 3.90 (3H, s), 7.25 (1H, t, *J* = 4.8 Hz), 7.32–7.40 (2H, m), 7.68 (1H, d, *J* = 8.7 Hz), 7.98 (1H, d, *J* = 8.4 Hz).

^**13**^ **C NMR (75 MHz, CDCl**_**3**_ **) δ:** 16.0, 19.7, 61.3, 120.1, 120.2, 125.7, 126.0, 126.2, 128.3, 129.2, 133.0, 134.6, 154.0.

**HRMS [ESI­(+)]** calcd. for [C_13_H_14_O_2_+H]^+^ 187.1117, found 187.1111.

**IR (film):** 3067, 3033, 2937, 2859, 2839, 2014, 1901, 1625, 1599, 1579, 1511, 1470, 1450, 1408, 1382, 1366, 1332, 1245, 1205, 1175, 1163, 1148, 1072, 1035, 1018, 990, 934, 869, 832, 818, 799, 751, 709 cm^–1^.

### 5-Methoxy-1,6-dimethyl-1,2-dihydronaphthalene (**27**)


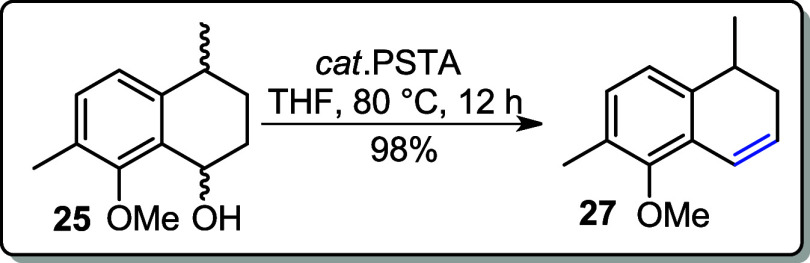


The reaction was performed following the general protocol as described for the synthesis of **26** but with the reflux temperature decreased to 80 °C for 12 h.

**Yield:** 98% (0.184 g, 0.98 mmol).

**Sample appearance:** colorless oil.

^**1**^ **H NMR (300 MHz, CDCl**_**3**_ **) δ:** 1.21 (3H, d, *J* = 6.9 Hz), 2.03–2.13 (1H, m), 2.25 (3H, s), 2.38–2.48 (1H, m), 2.82–2.94 (1H, m), 3.71 (3H, s), 5.99 (1H, q, *J* = 4.6 Hz), 6.76 (1H, d, *J* = 9.9 Hz), 6.85 (1H, d, *J* = 7.5 Hz), 6.98 (1H, d, *J* = 7.5 Hz).

^**13**^ **C NMR (75 MHz, CDCl**_**3**_ **) δ:** 15.9, 20.3, 31.2, 31.7, 61.0, 121.8, 121.9, 126.3, 127.6, 128.7, 129.4, 140.2, 154.3.

**HRMS [ESI­(+)]** calcd. for [C_13_H_16_O+Na]^+^ 211.1099, found 211.1092.

**IR (film):** 3311, 3070, 3039, 2925, 2861, 1625, 1599, 1579, 1511, 1471, 1408, 1382, 1366, 1245, 1205, 1163, 1018, 990, 799, 752 cm^–1^.

### (1*R*,2*R*)-1,2,8-Trimethoxy-4,7-dimethyl-1,2,3,4-tetrahydronaphthalene (**28**) and (1*S,*2*R*)-1,2,8-trimethoxy-4,7-dimethyl-1,2,3,4-tetrahydronaphthalene (**29**)


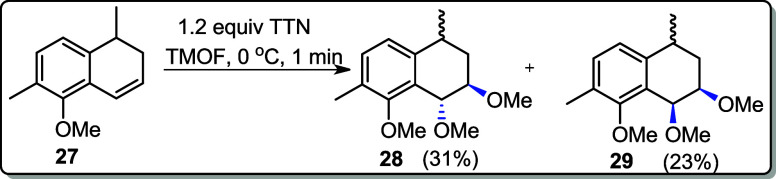


To a stirred solution of alkene **27** (0.049 g, 0.26 mmol) in TMOF (2 mL) was added TTN (0.139 g, 0.312 mmol) at 0 °C. The mixture was stirred for 1 min, which led to an abundant white precipitate. The resulting suspension was filtered through a silica gel pad using CH_2_Cl_2_ as the eluent. The filtrated solution was washed with H_2_O (2 × 10 mL) and with brine (1 × 10 mL) and dried over anhydrous MgSO_4_. The solvent was removed at reduced pressure, and the crude was purified by flash chromatography (10% EtOAc in hexane).

#### (1*R*,2*R*)**-**1,2,8-Trimethoxy-4,7-dimethyl-1,2,3,4-tetrahydronaphthalene **(27)**

**Yield:** 31% (0.020 g, 0.08 mmol).

**Sample appearance:** light yellow oil.

^**1**^ **H NMR (300 MHz, CDCl**_**3**_ **) δ:** 1.33 (3H, d, *J* = 7.2 Hz), 1.48–1.57 (1H, q, *J* = 6.6 Hz), 2.21–2.28 (4H, m), 2.88–3.00 (1H, m), 3.40 (3H, s), 3.42 (3H, s), 3.79 (3H, s), 3.84–3.88 (1H, m), 4.65 (1H, d, *J* = 2.7 Hz), 6.93 (1H, d, *J* = 7.8 Hz), 7.10 (1H, d, *J* = 7.8 Hz).

^**13**^ **C NMR (75 MHz, CDCl**_**3**_ **) δ:** 16.1, 22.8, 30.6, 33.1, 56.6, 59.2, 61.0, 71.6, 81.0, 123.2, 128.1, 128.9, 131.5, 140.9, 157.3.

**HRMS [ESI­(+)]** calcd. for [C_15_H_22_O_3_+Na]^+^ 273.1461, found 273.1451.

**IR (film):** 3351, 2932, 2872, 2826, 1719, 1609, 1574, 1453, 1433, 1369, 1359, 1255, 1077, 817 cm^–1^.

#### (1*S*,1*R*)-1,2,8-Trimethoxy-4,7-dimethyl-1,2,3,4-tetrahydronaphthalene **(28)**

**Yield:** 23% (0.014 g, 0.06 mmol).

**Sample appearance:** light yellow oil.

^**1**^ **H NMR (500 MHz, CDCl**_**3**_ **) δ:** 1.35 (3H, d, *J* = 6.9 Hz), 1.85–1.97 (1H, m), 2.00–2.07 (1H, m), 2.29 (3H, s), 2.82–2.95 (1H, m), 3.36–3.43 (1H, dt, *J* = 3.7 Hz), 3.49 (3H, s), 3.61 (3H, s), 3.80 (3H, s), 4.82 (1H, d, *J* = 4.2 Hz), 6.98 (1H, d, *J* = 8.1 Hz), 7.11 (1H, d, *J* = 8.1 Hz).

^**13**^ **C NMR (75 MHz, CDCl**_**3**_ **) δ:** 16.1, 22.3, 29.6, 32.6, 56.6, 56.9, 61.2, 74.2, 78.3, 122.3, 127.6, 128.1, 131.1, 142.4, 158.1.

**HRMS [ESI­(+)]** calcd. for [C_15_H_22_O_3_+Na]^+^ 273.1461, found 273.1458.

**IR (film):** 3368, 2931, 2826, 2872, 1733, 1669, 1608, 1574, 1488, 1433, 1369, 1255, 1094, 1078, 816 cm^–1^.

### (3*R*)-7-Methoxy-3,6-dimethyl-2,3-dihydro-1*H*-indene-1-carbaldehyde (±)­(**30**)


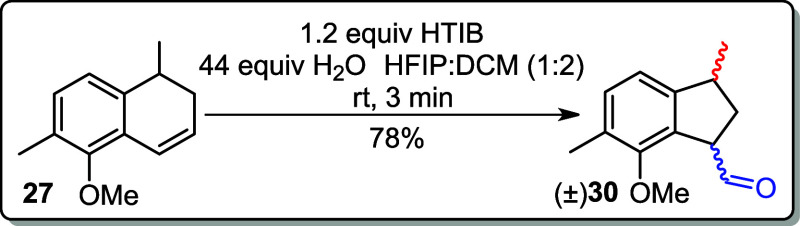


To a stirred solution of alkene **27** (0.030 g, 0.16 mmol) in HFIP/DCM (1:2) (1.5 mL) and H_2_O (0.126 g, 44 equiv) was added HTIB (0.075 g, 0.20 mmol) at rt. This mixture was stirred for 3 min. The reaction was quenched with a saturated solution of NaHCO_3_ and extracted with EtOAc (3 × 10 mL). The combined organic layers were washed with brine (10 mL) and dried over anhydrous MgSO_4_. The solvent was removed at reduced pressure, and the crude was purified by flash chromatography (5% EtOAc in hexane).

**Yield:** 78% (0.025 g, 0.12 mmol).

**Sample appearance:** colorless oil.

^**1**^ **H NMR (300 MHz, CDCl**_**3**_ **) δ:** 1.27 (3H, d, *J* = 6.6 Hz), 1.75–1.88 (1H, m), 2.23 (3H, s), 2.57–2.66 (1H, m), 3.22–3.32 (1H, m), 3.76 (3H, s), 3.87–4.11 (1H, m), 6.91 (1H, d, *J* = 7.8 Hz), 7.11 (1H, d, *J* = 7.8 Hz), 9.67 (1H, d, *J* = 2.7 Hz).

^**13**^ **C NMR (75 MHz, CDCl**_**3**_ **) δ:** 16.0, 20.6, 35.3, 38.7, 54.7, 60.2, 119.5, 129.0, 130.8, 131.9, 149.6, 155.5, 201.0.

**HRMS [ESI­(+)]** calcd. for [C_13_H_16_O_2_+Na]^+^ 227.1043, found 2273.1012.

**IR (film):** 3071, 3029, 2928, 2839, 1731, 1699, 1638, 1599, 1511, 1475, 1408, 1382, 1366, 1240, 1171, 1162, 1145, 1070, 1035, 1018, 999, 870, 832, 819, 799, 755, 714 cm^–1^.

### (3*R*)-7-Methoxy-3,6-dimethyl-2,3-dihydro-1*H*-indene-1-carbaldehyde (±)­(**30**)


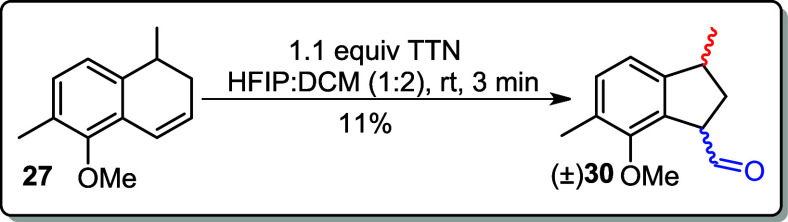


The reaction was performed following the general protocol as described above; using alkene **27** (0.030 g, 0.16 mmol) in HFIP/DCM (1:2) (1.5 mL), TTN (0.078 g, 0.18 mmol) was added at rt. The crude product was purified by flash column chromatography (5% EtOAc in hexane), giving ±**(30)** (0.004 g, 0.02 mmol, 11%).

^**1**^ **H NMR (300 MHz, CDCl**_**3**_ **) δ:** data given above.

^**13**^ **C NMR (75 MHz, CDCl**_**3**_ **) δ:** data given above.

## Supplementary Material



## Notes

Schematic representation toward the total synthesis of (±)-jungianol (Scheme S1); ^1^H NMR and ^13^C NMR analysis for all novel synthesized compounds (Figure S1–S26) (PDF)

## Notes

The authors express their heartfelt gratitude to their late supervisor, Prof. Dr. Luiz Fernando da Silva Jr. (1971–June 7, 2017), in loving memory.
