# Supplementary material for: Toward the Total Synthesis of Sesquiterpene via an Annulative and Oxidative Approach
Source: ACS Omega. 2025 Jul 25;10(30):33240–52. doi: 10.1021/acsomega.5c03127 (PMC12332555; doi:10.1021/acsomega.5c03127)

Supporting information

for

Toward the Total Synthesis of Sesquiterpene via an Annulative and Oxidative Approach

Ajmir Khan*[a,b] and Fernando C. Rezende[b]

*Corresponding author: khanajmi@msu.edu

[a] School of Packaging, Michigan State University, 448 Wilson Rd, East Lansing, MI, USA

[b] Department of Fundamental Chemistry,
Institute of Chemistry, University of São Paulo, Av. Prof. Lineu Prestes, 748, São Paulo, SP CEP 05508-000, Brazil

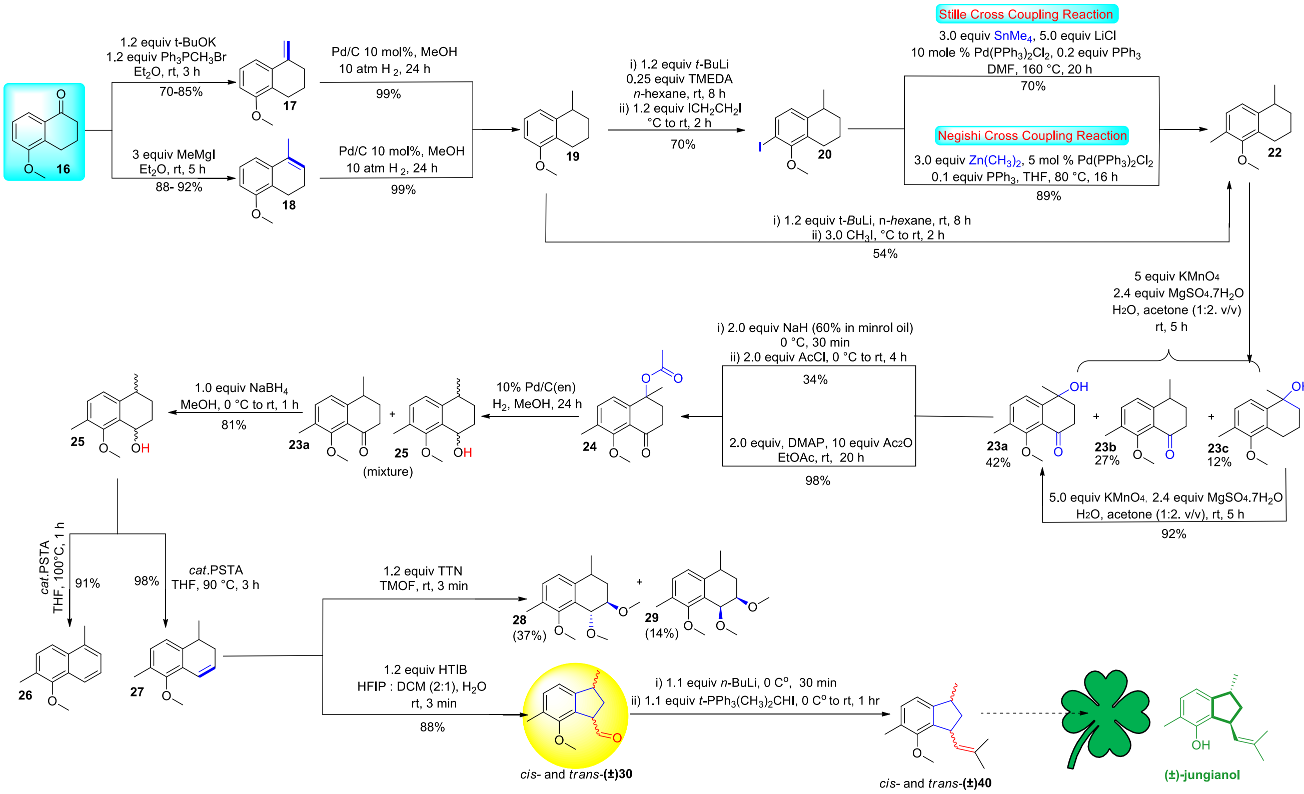

Scheme S1. Schematic Representation towards the total synthesis of *(±)*-Jungianol

Figure S1: 6-Iodo-5-methoxy-1-methyl-1,2,3,4-tetrahydronaphthalene (20).

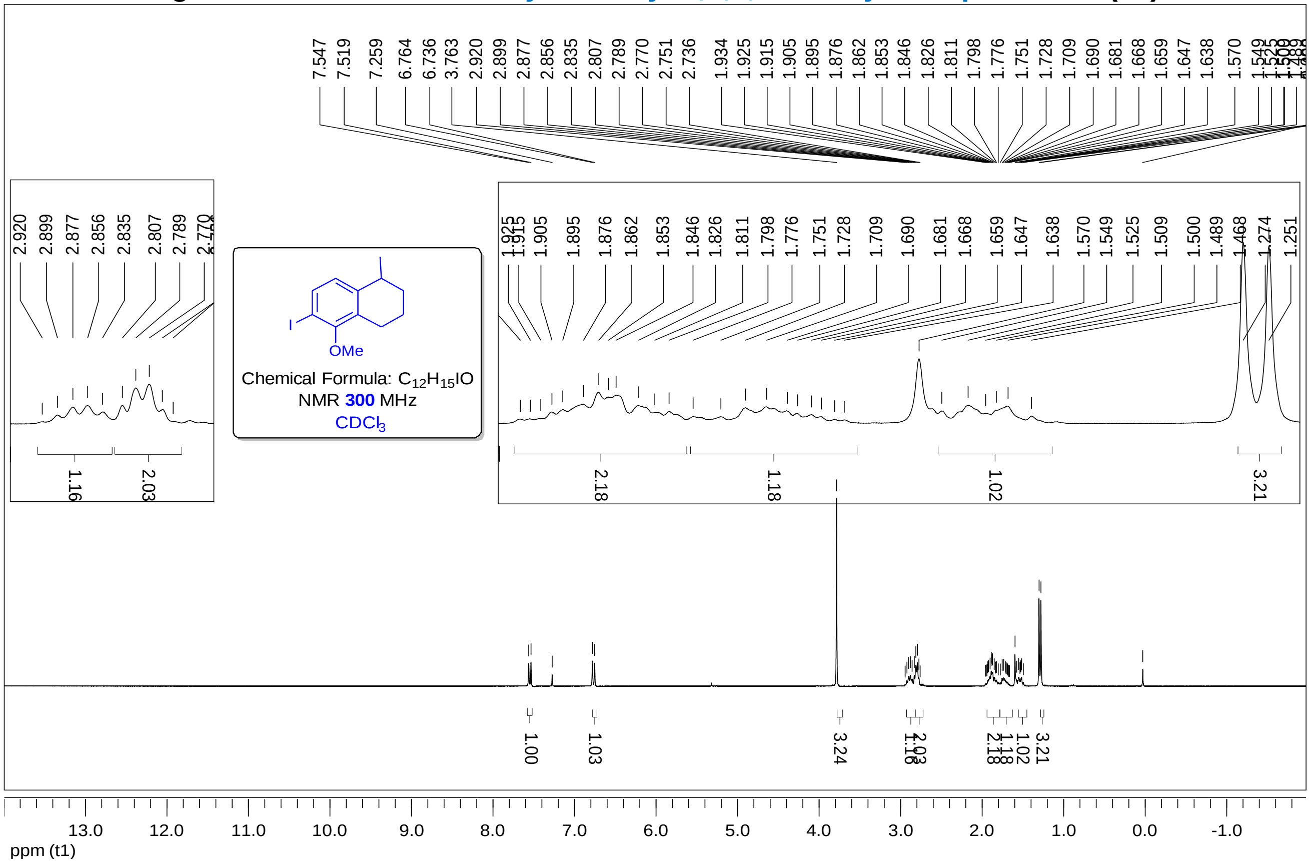

Figure S2: 6-Iodo-5-methoxy-1-methyl-1,2,3,4-tetrahydronaphthalene (20).

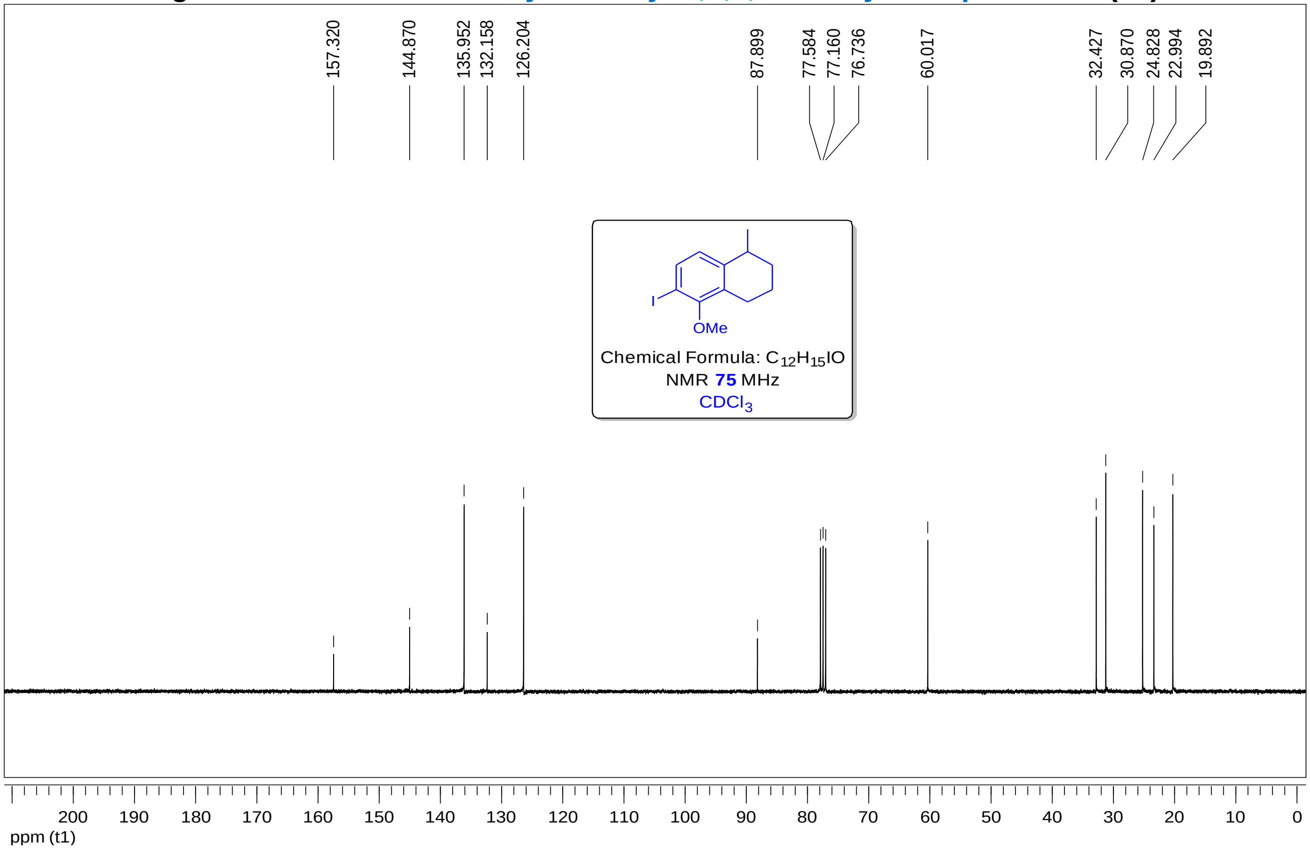

Figure S3: 1-Methoxy-5-methyl-5,6,7,8-tetrahydronaphthalen-2-ol (21)

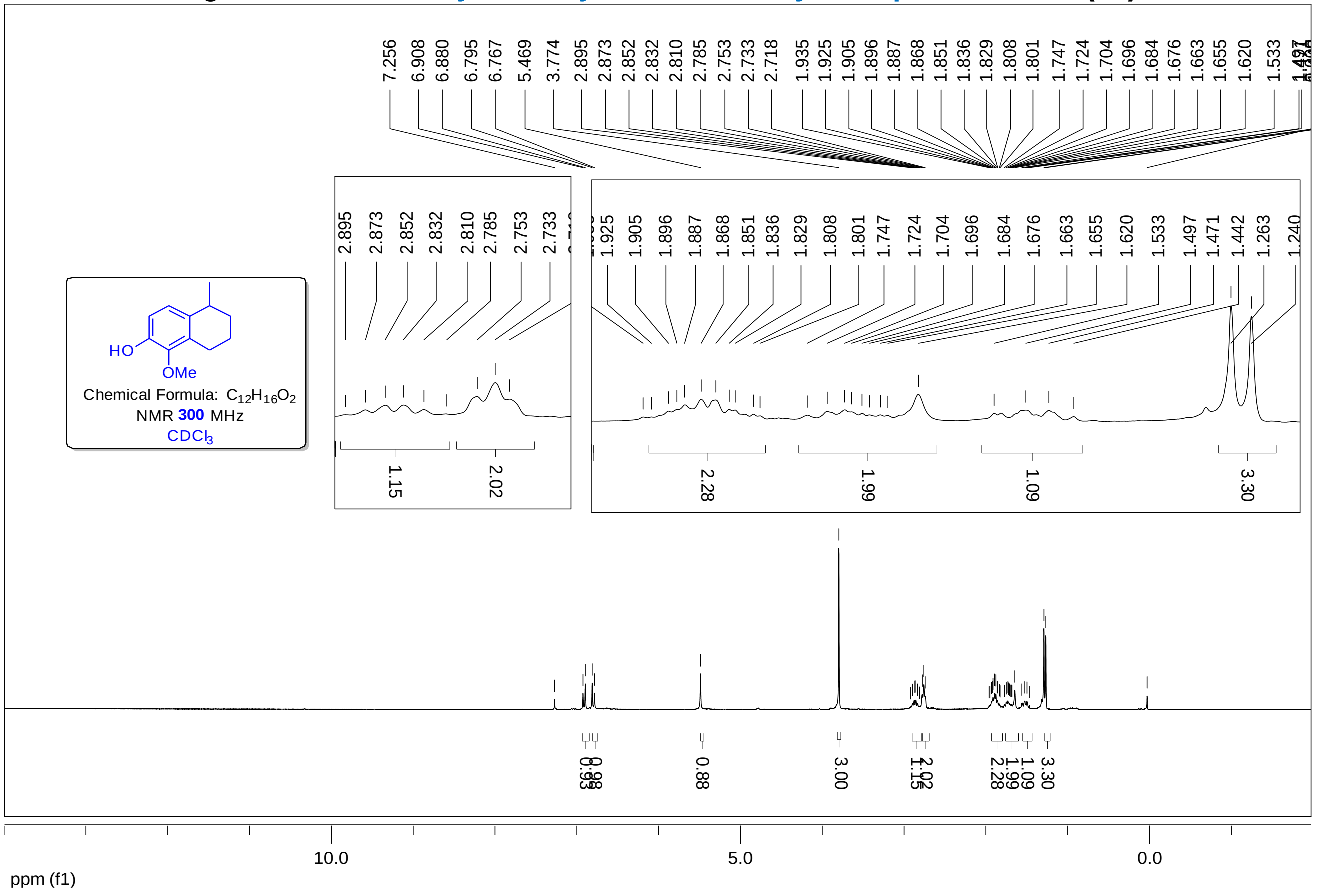

Figure S4: 1-Methoxy-5-methyl-5,6,7,8-tetrahydronaphthalen-2-ol (21).

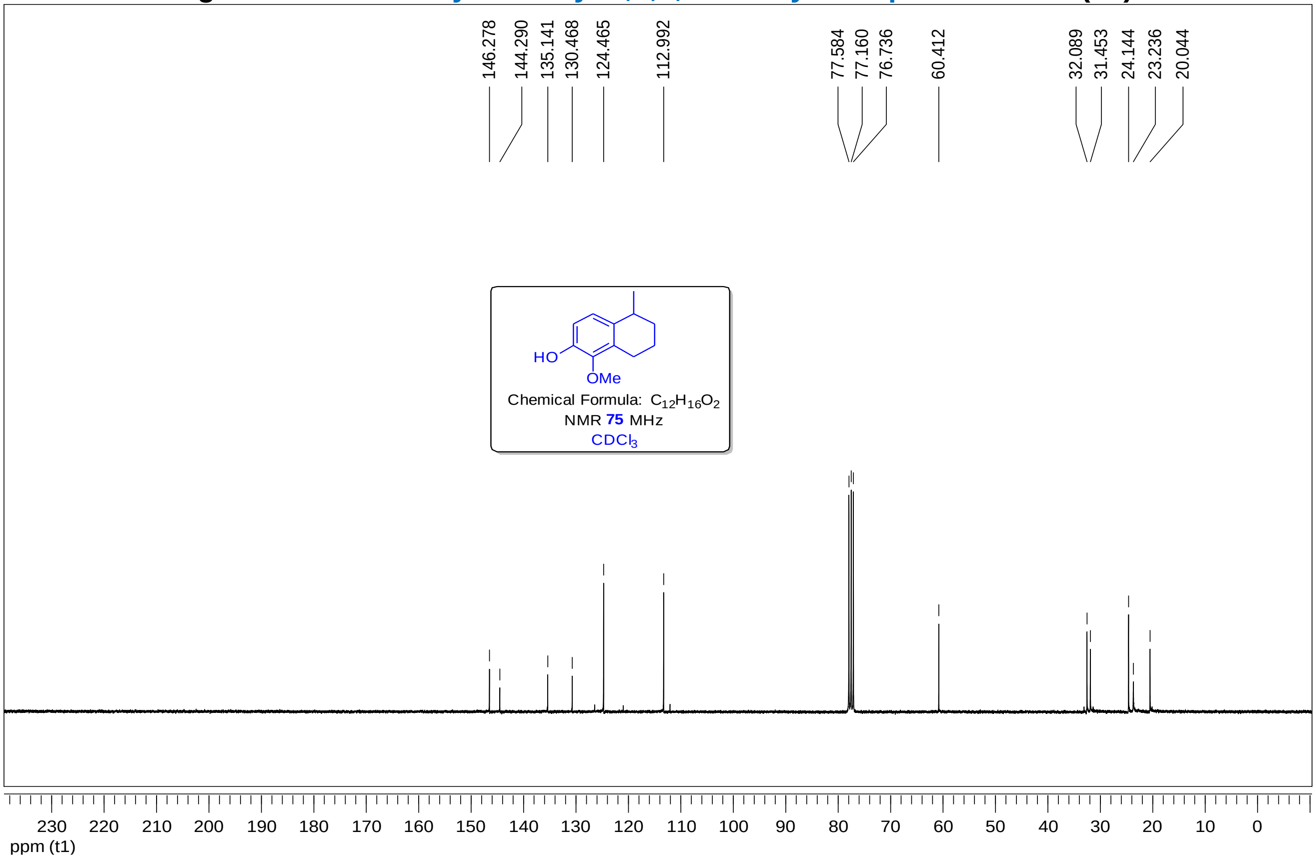

Figure S5: 5-Methoxy-1,6-dimethyl-1,2,3,4-tetrahydronaphthalene (22).

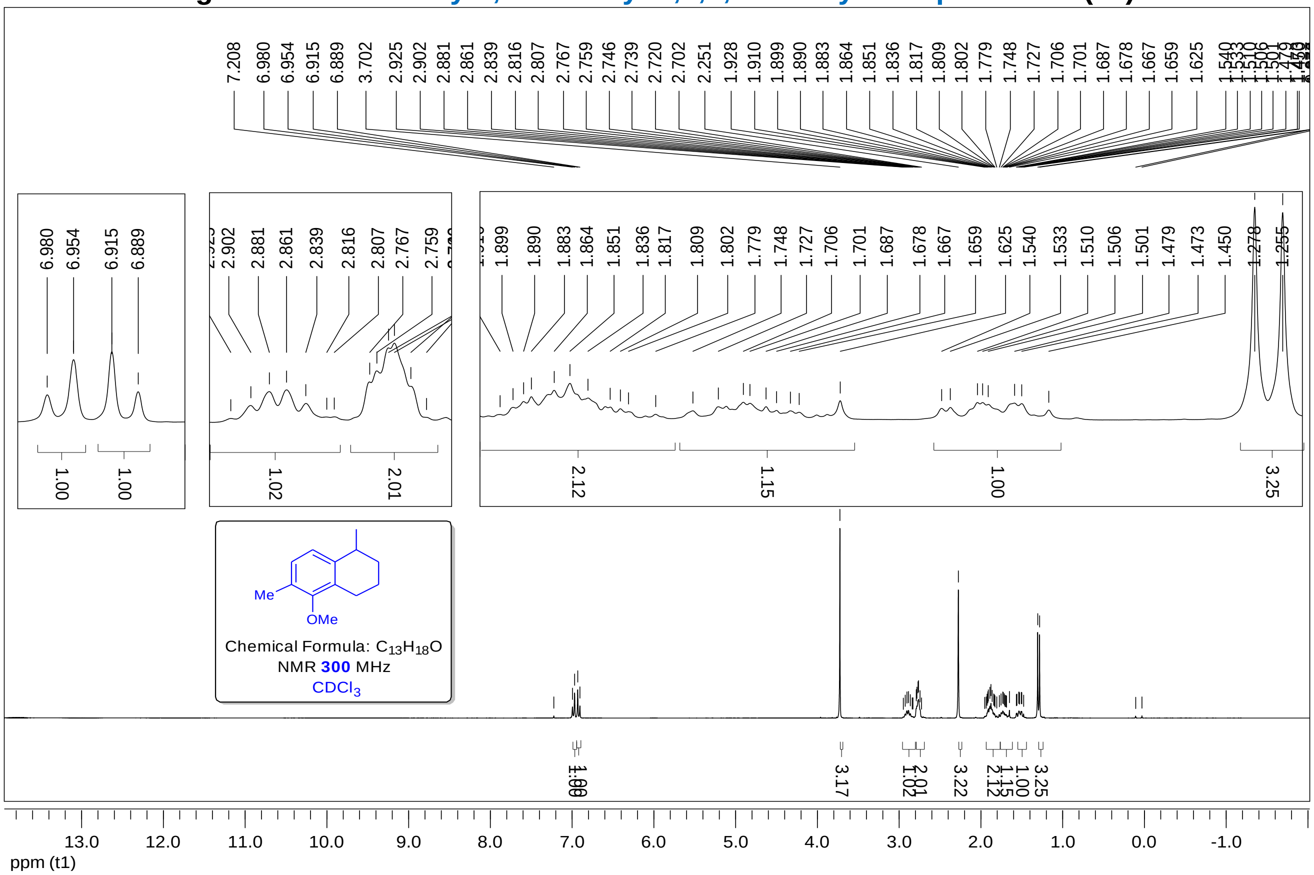

Figure S6: 5-Methoxy-1,6-dimethyl-1,2,3,4-tetrahydronaphthalene (22).

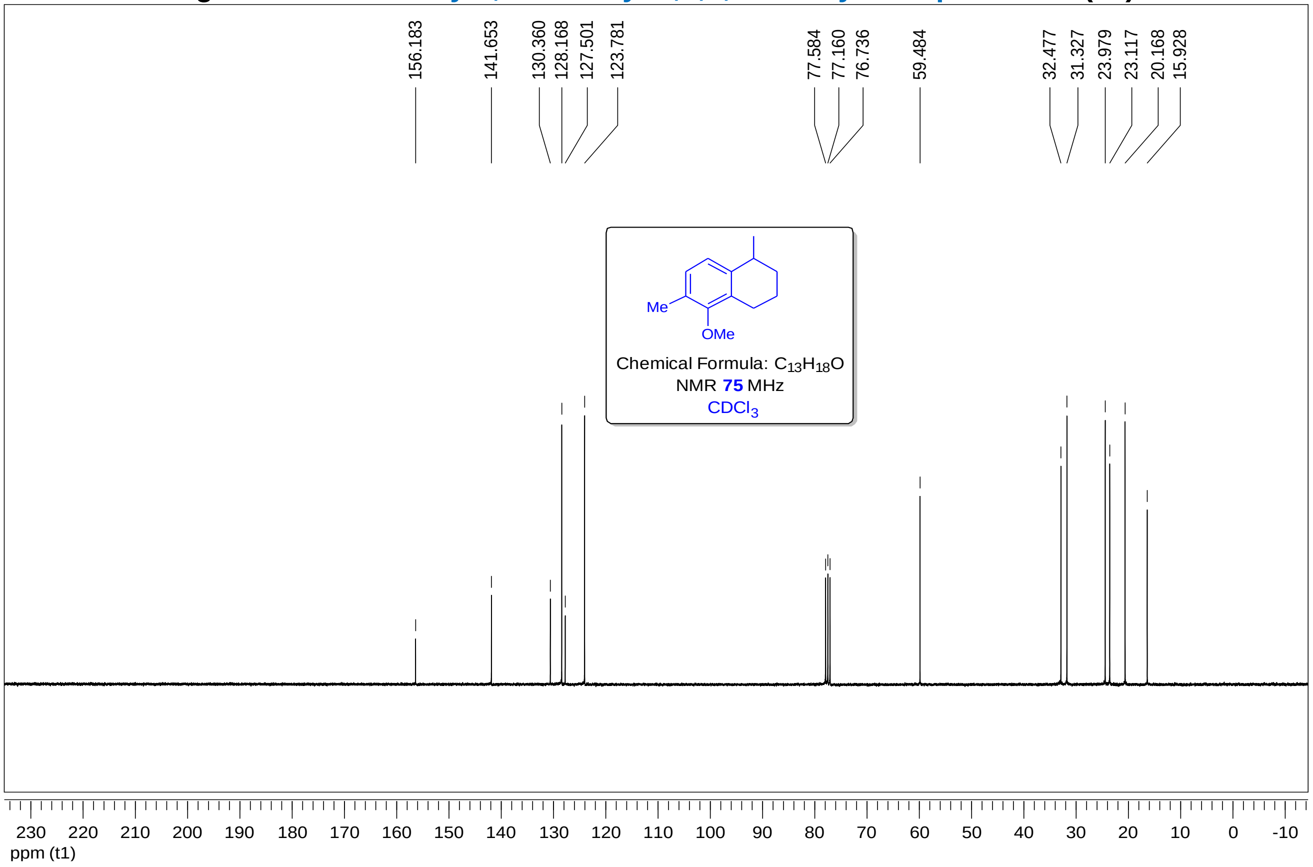

Figure S7: 4-Hydroxy-8-methoxy-4,7-dimethyl-3,4-dihydronaphthalen-1(2*H*)-one (23a).

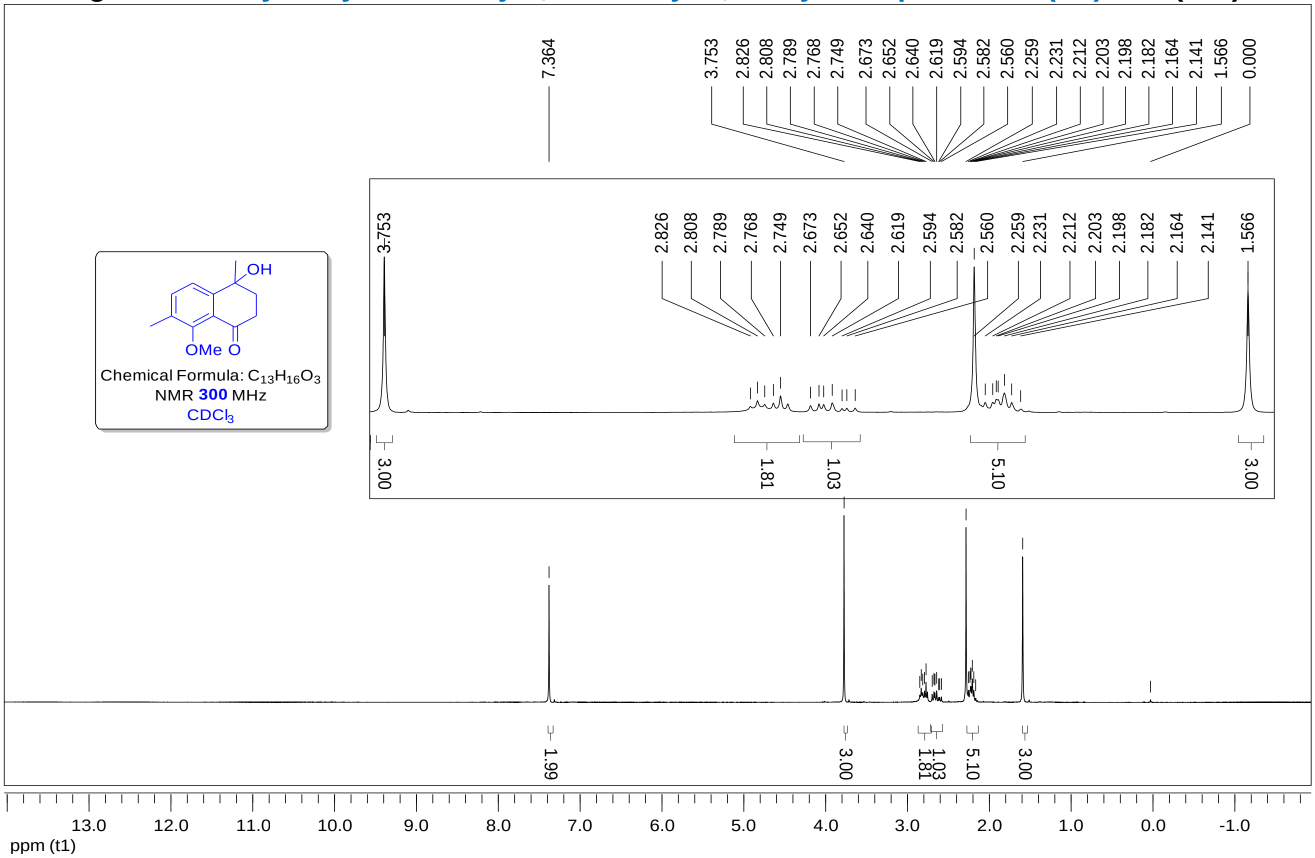

Figure S8: 4-Hydroxy-8-methoxy-4,7-dimethyl-3,4-dihydronaphthalen-1(2*H*)-one (23a).

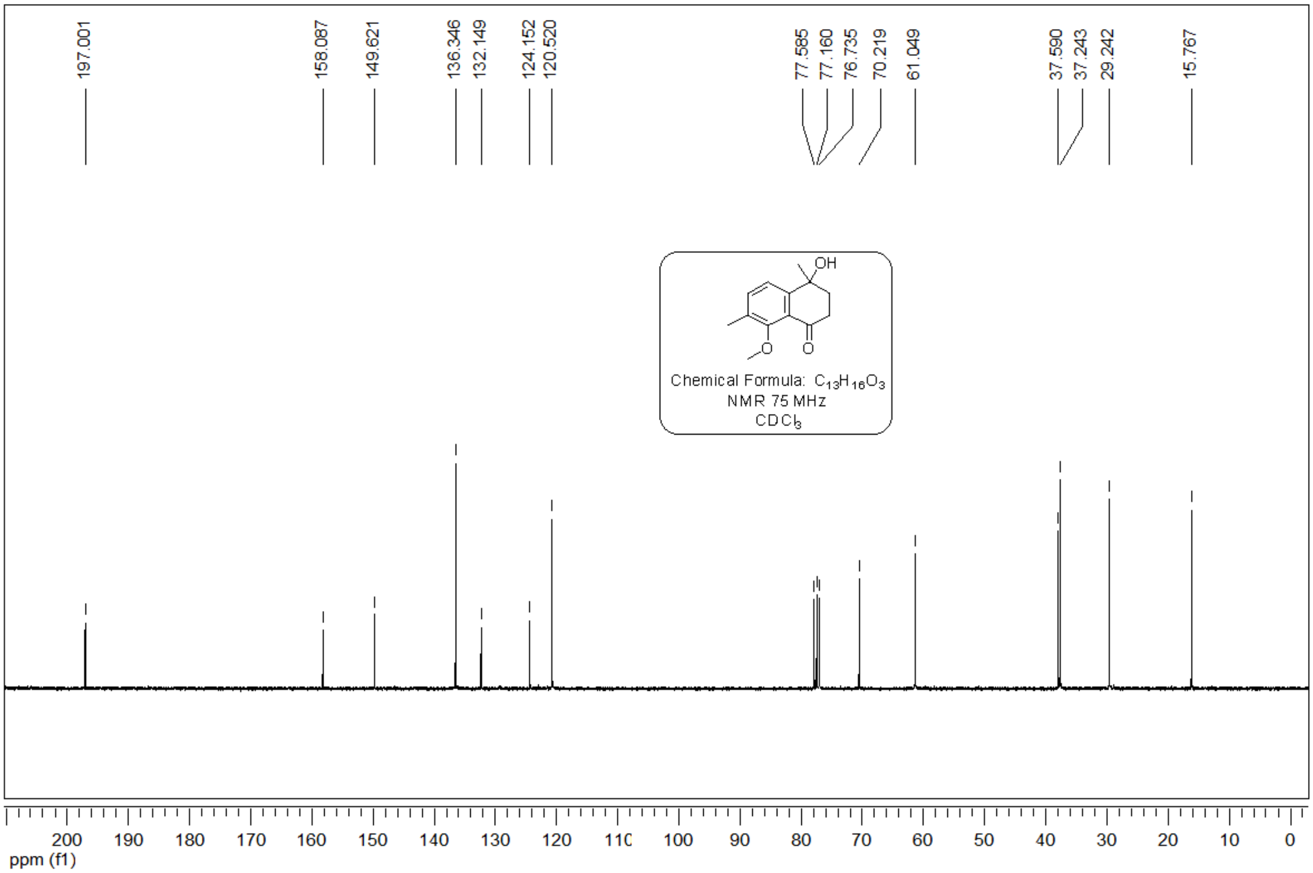

Figure S9: 8-Methoxy-4,7-dimethyl-3,4-dihydronaphthalen-1(2*H*)-one (23b).

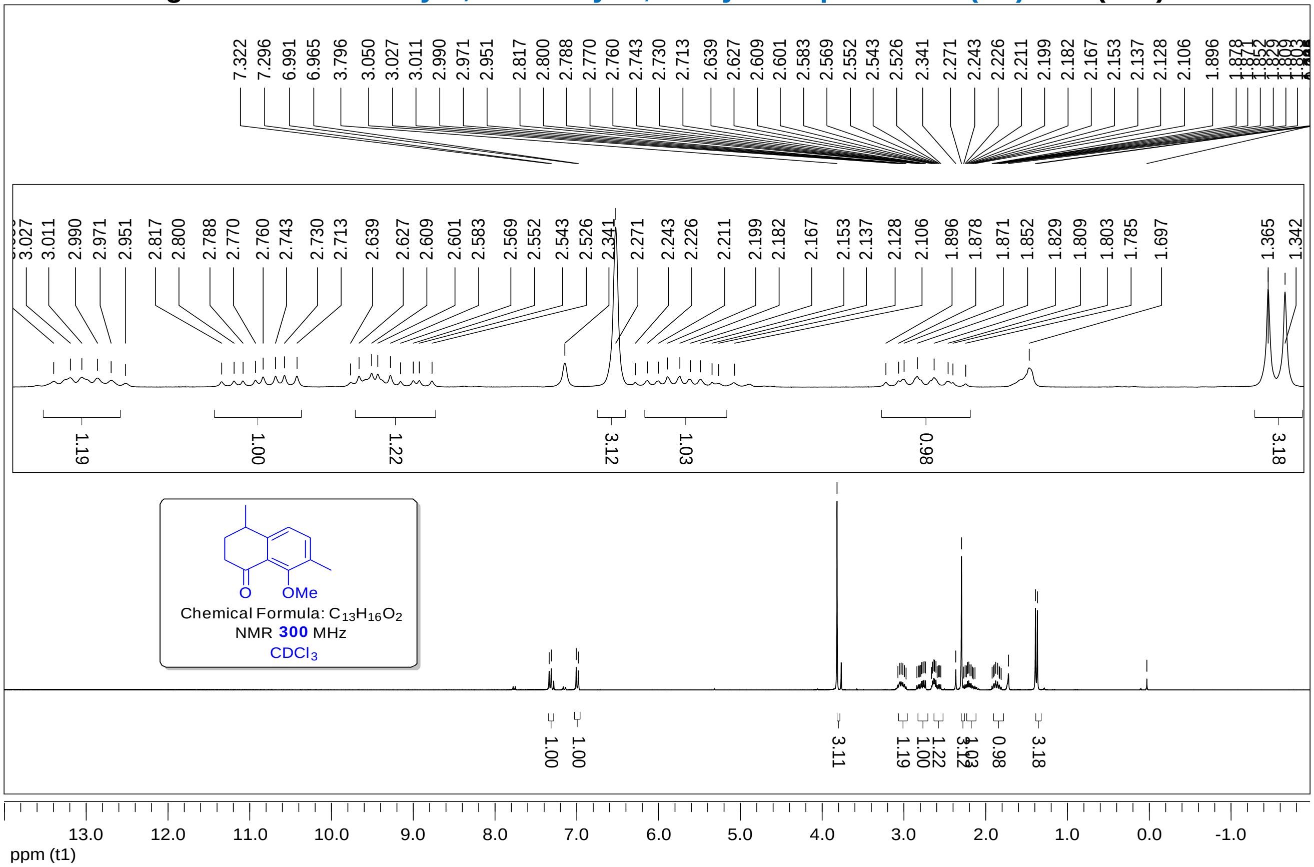

Figure S10: 8-Methoxy-4,7-dimethyl-3,4-dihydronaphthalen-1(2*H*)-one (23b).

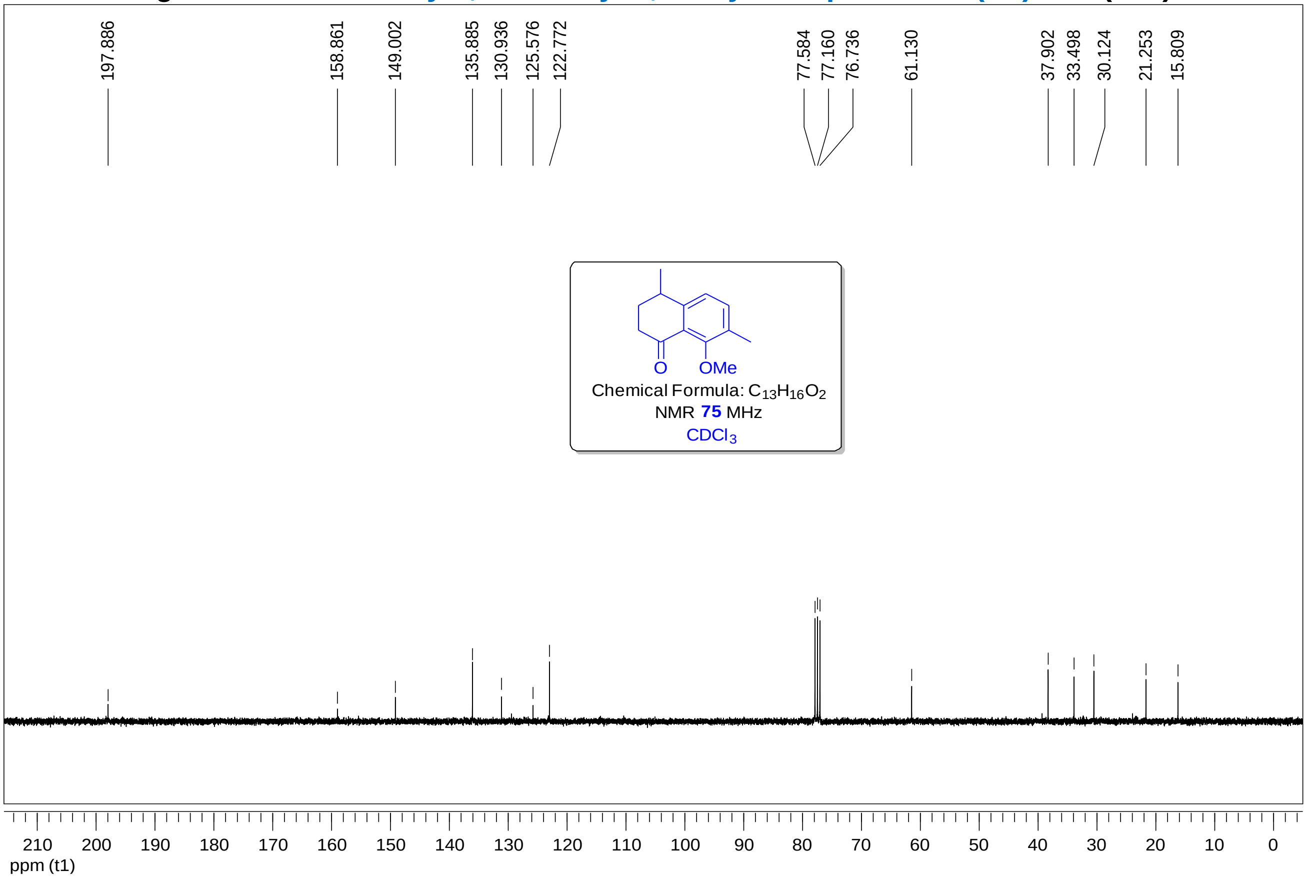

Figure S11: 5-Methoxy-1,6-dimethyl-1,2,3,4-tetrahydronaphthalen-1-ol (23c).

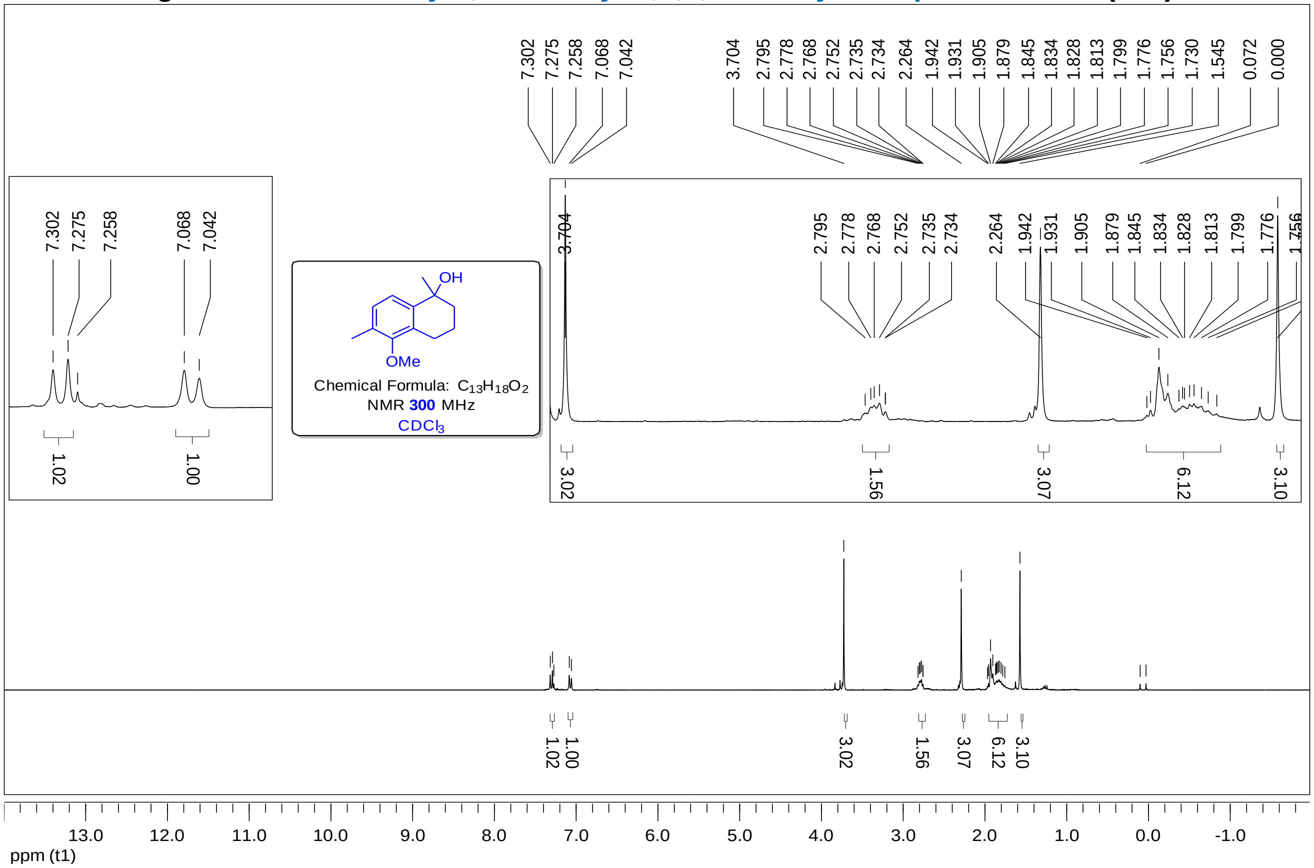

Figure S12: 5-Methoxy-1,6-dimethyl-1,2,3,4-tetrahydronaphthalen-1-ol (23c).

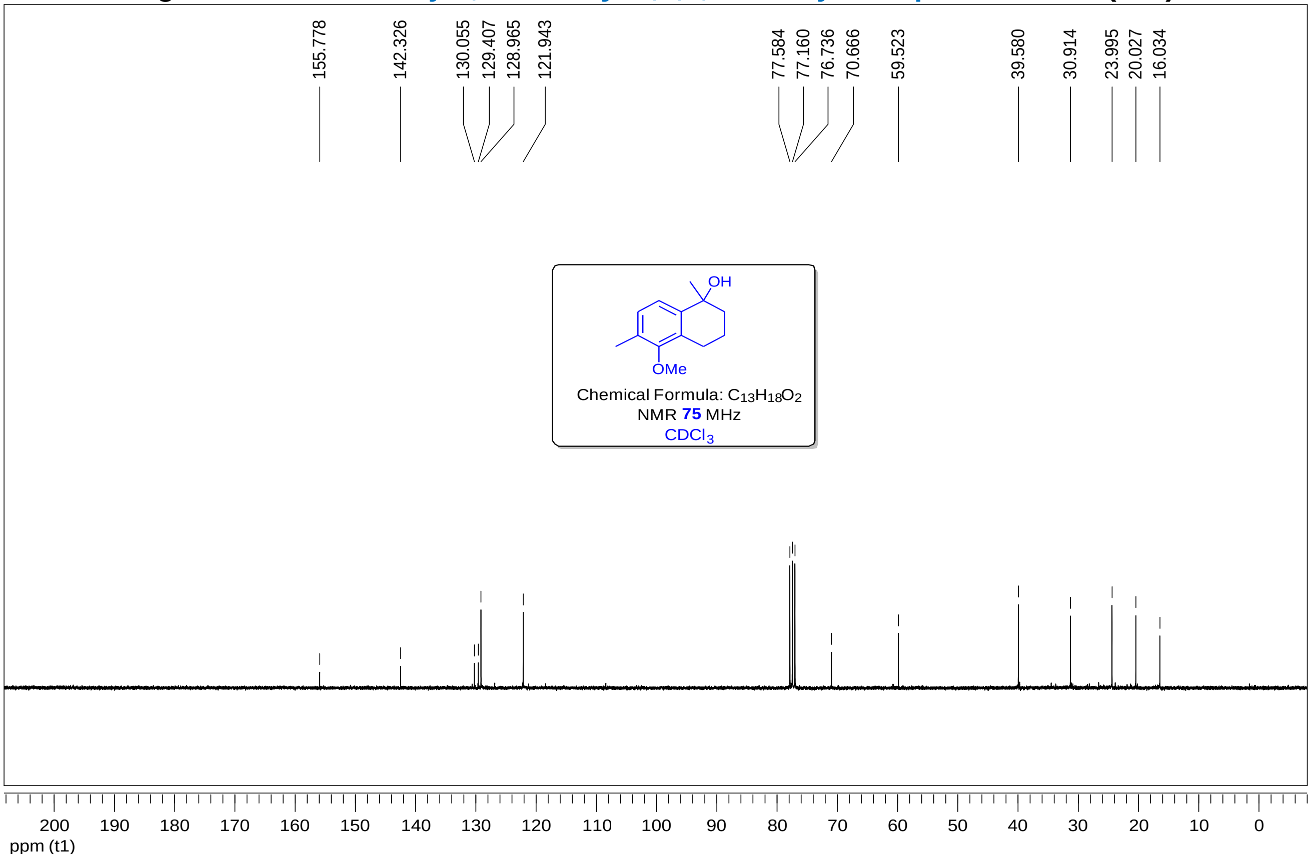

Figure S13: 5-Methoxy-1,6-dimethyl-4-oxo-1,2,3,4-tetrahydronaphthalen-1-yl acetate (24).

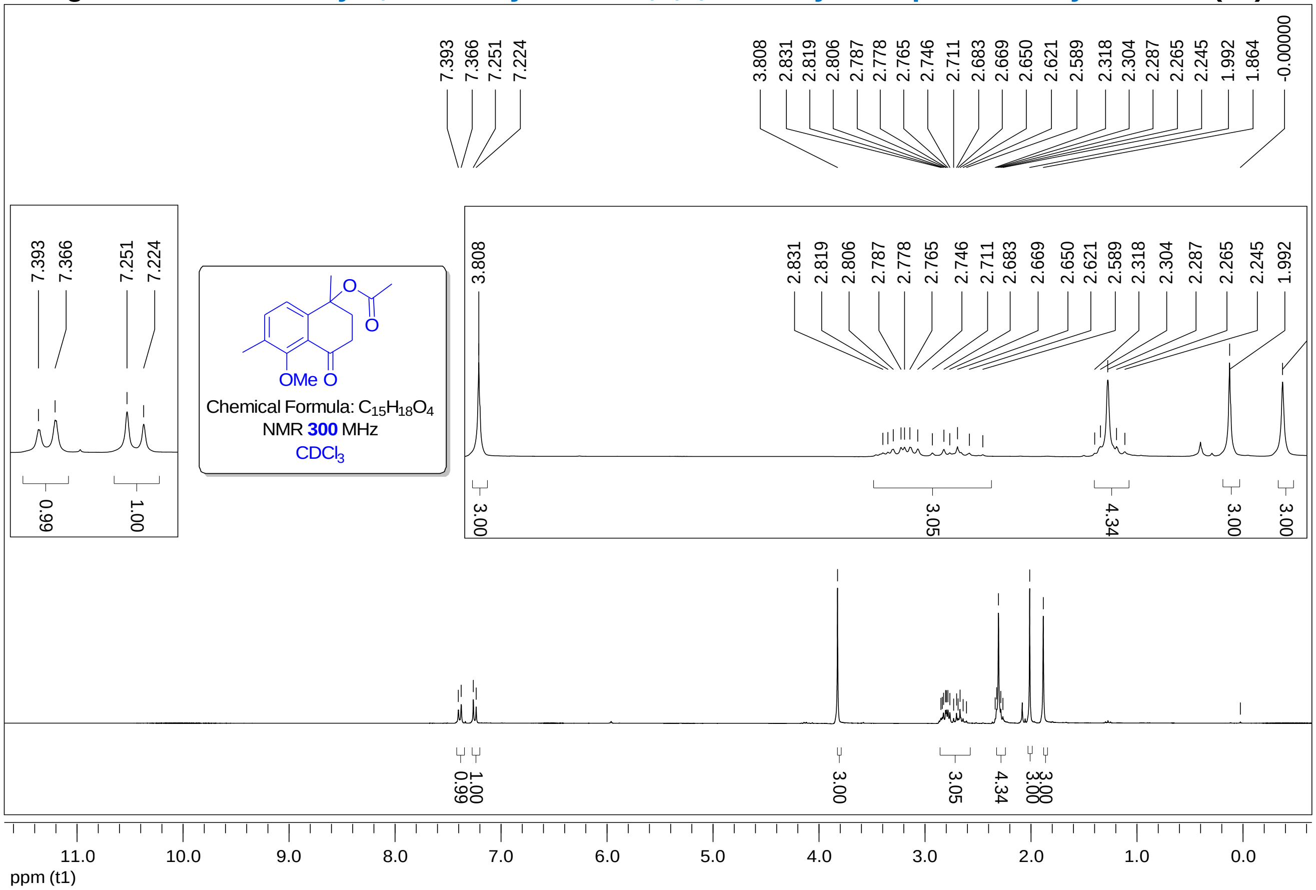

Figure S14: 5-Methoxy-1,6-dimethyl-4-oxo-1,2,3,4-tetrahydronaphthalen-1-yl acetate (24).

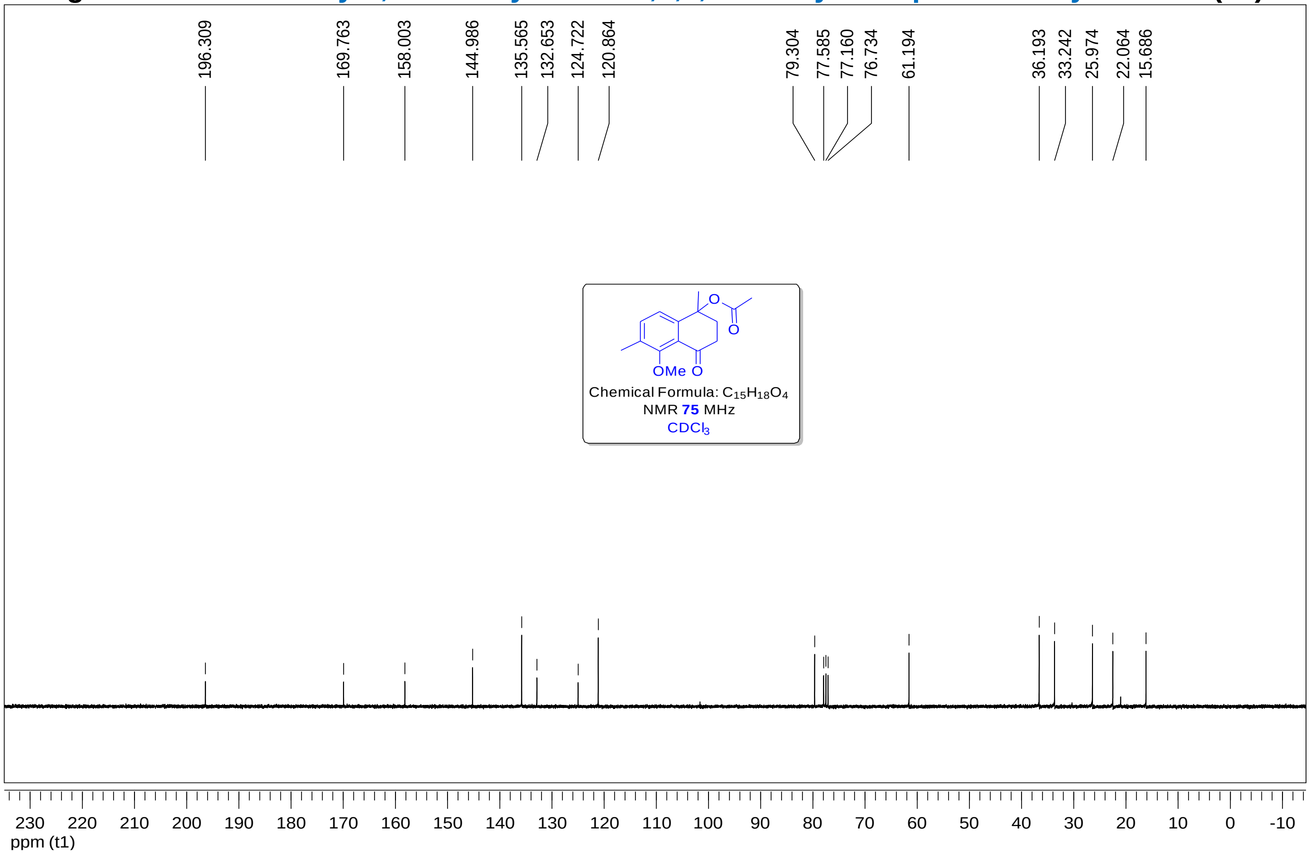

Figure S15: 8-Methoxy-4,7-dimethyl-1,2,3,4-tetrahydronaphthalen-1-ol (25).

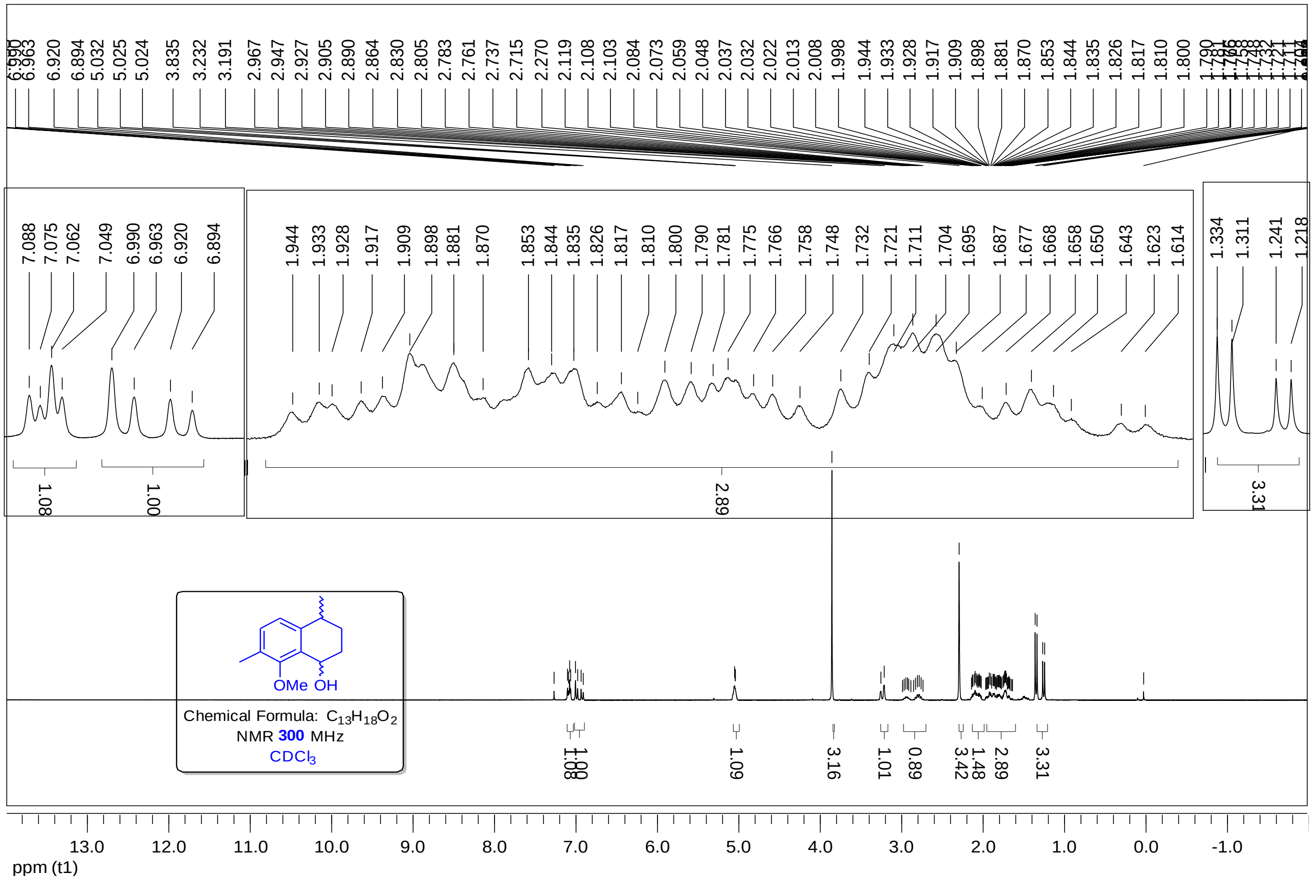

Figure S16: 8-Methoxy-4,7-dimethyl-1,2,3,4-tetrahydronaphthalen-1-ol (25).

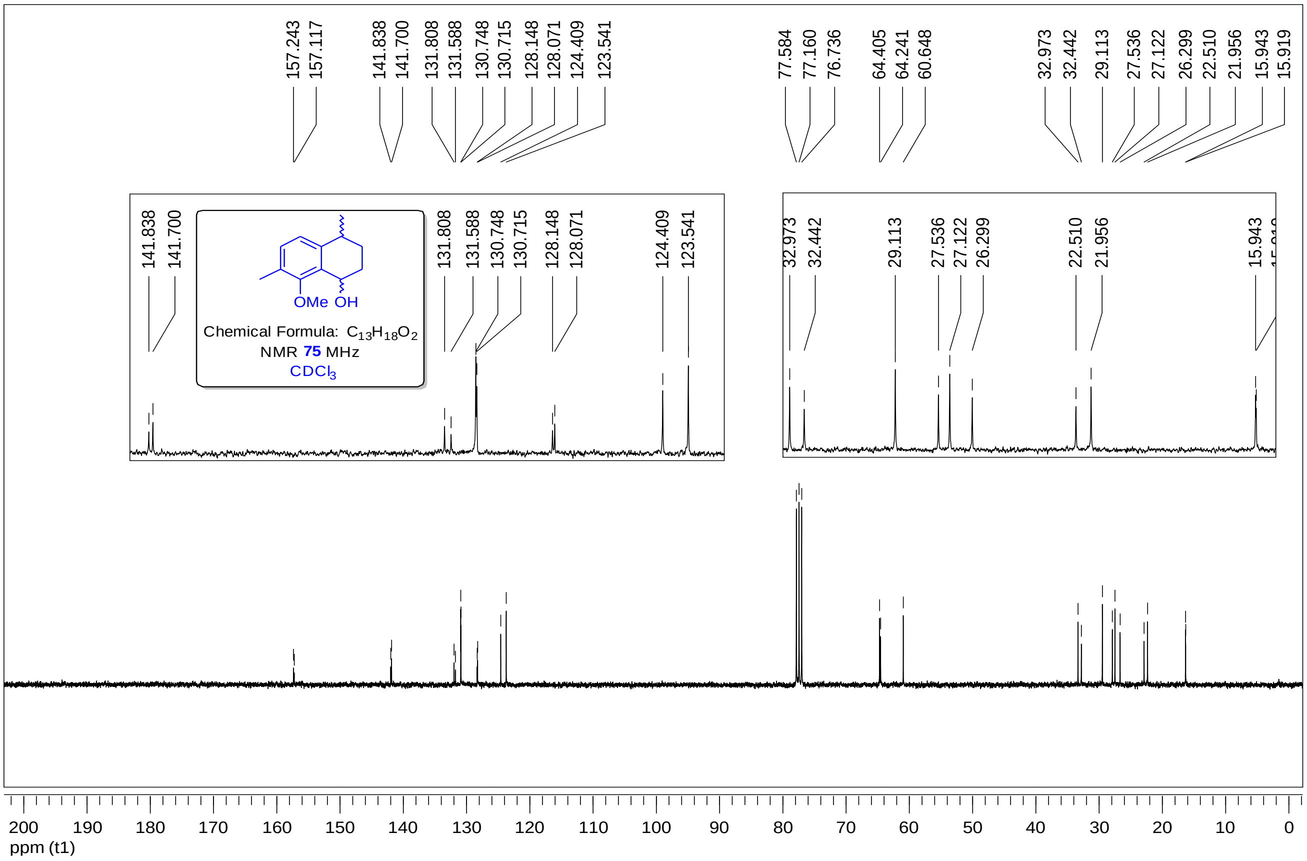

Figure S17: 81-Methoxy-2,5-dimethylnaphthalene (26).

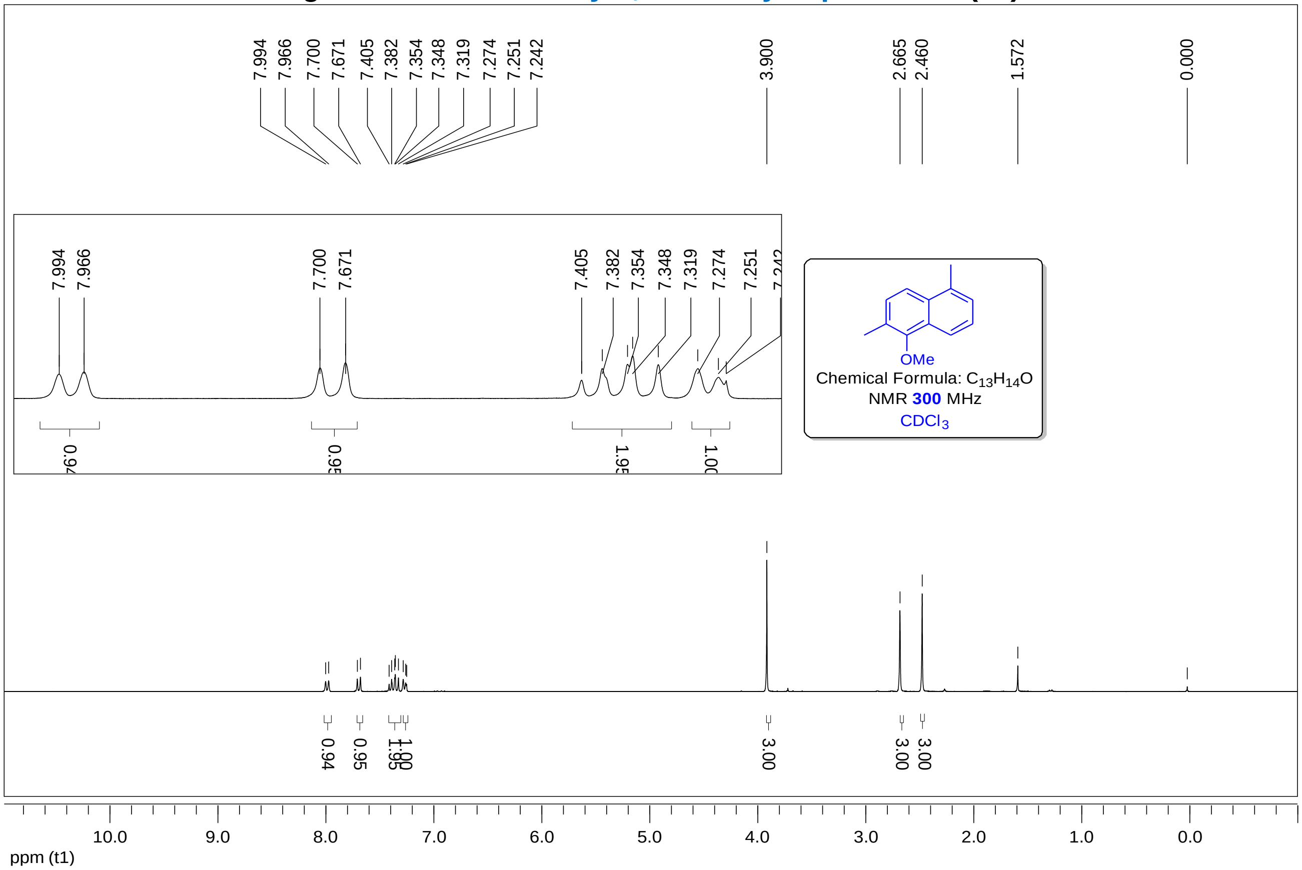

Figure S18: 1-Methoxy-2,5-dimethylnaphthalene (26).

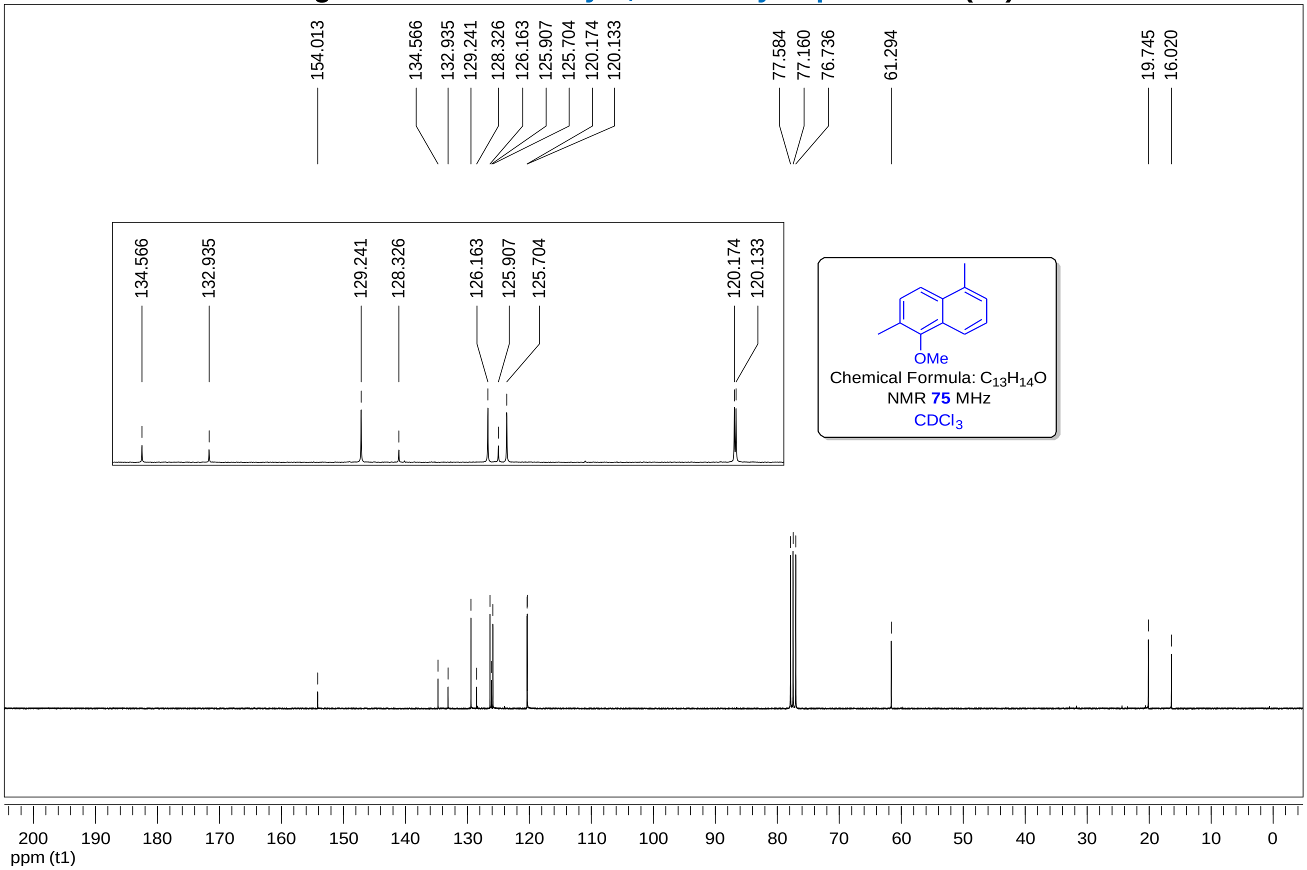

Figure S19: **5-Methoxy-1,6-dimethyl-1,2-dihydronaphthalene (27).**

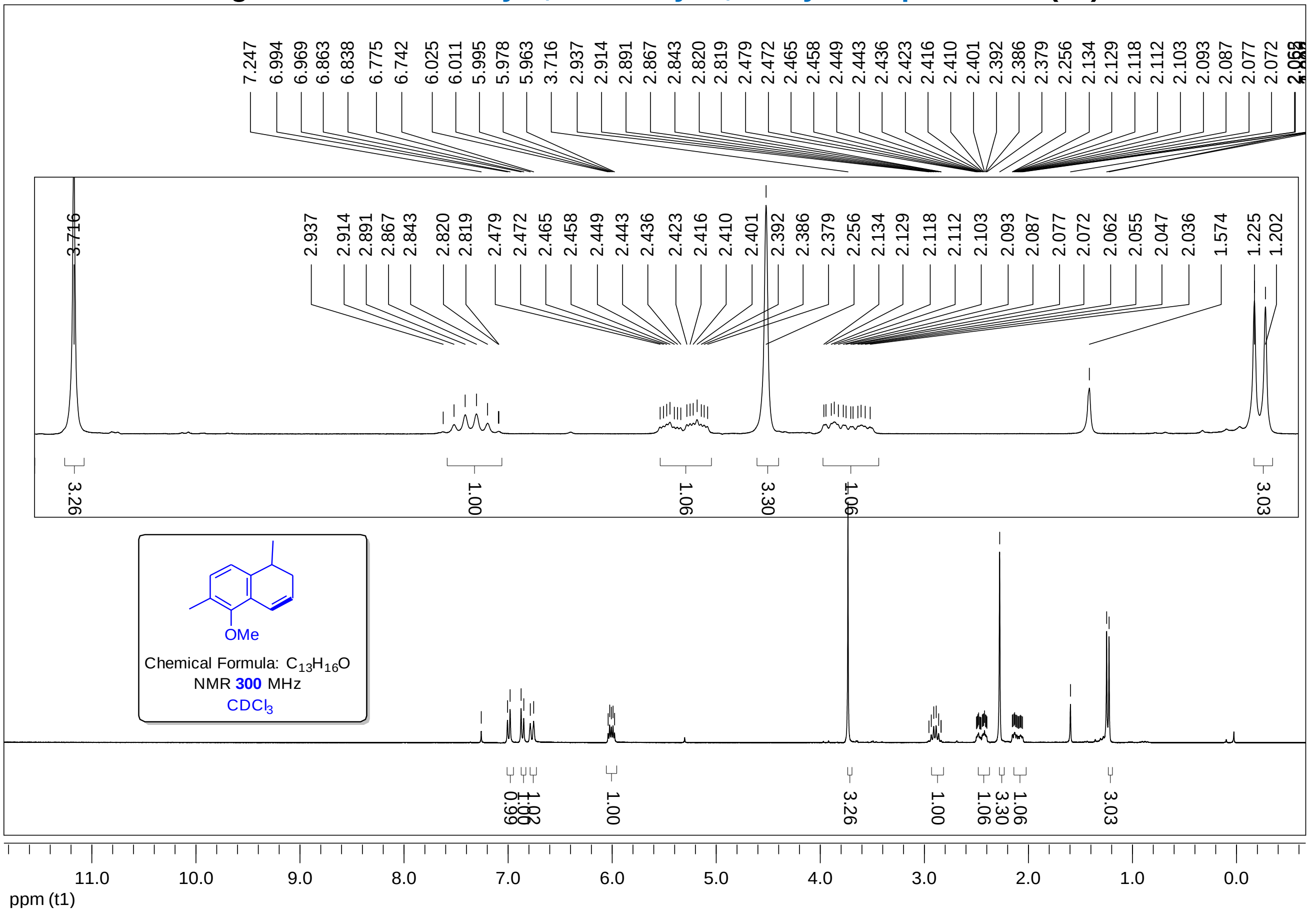

Figure S20: **5-Methoxy-1,6-dimethyl-1,2-dihydronaphthalene (27).**

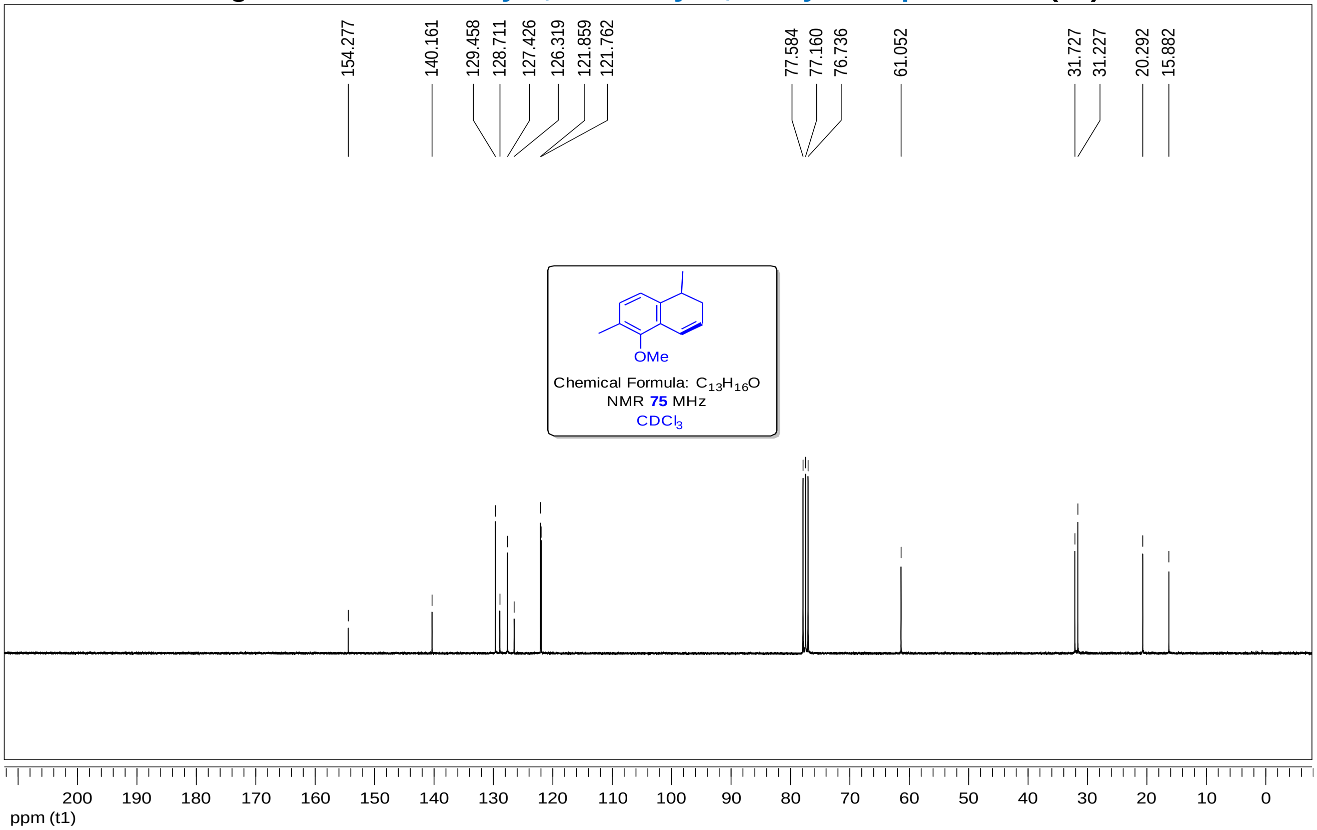

Figure S21: ***(1R,2R)*-1,2,8-Trimethoxy-4,7-dimethyl-1,2,3,4-tetrahydronaphthalene (28).**

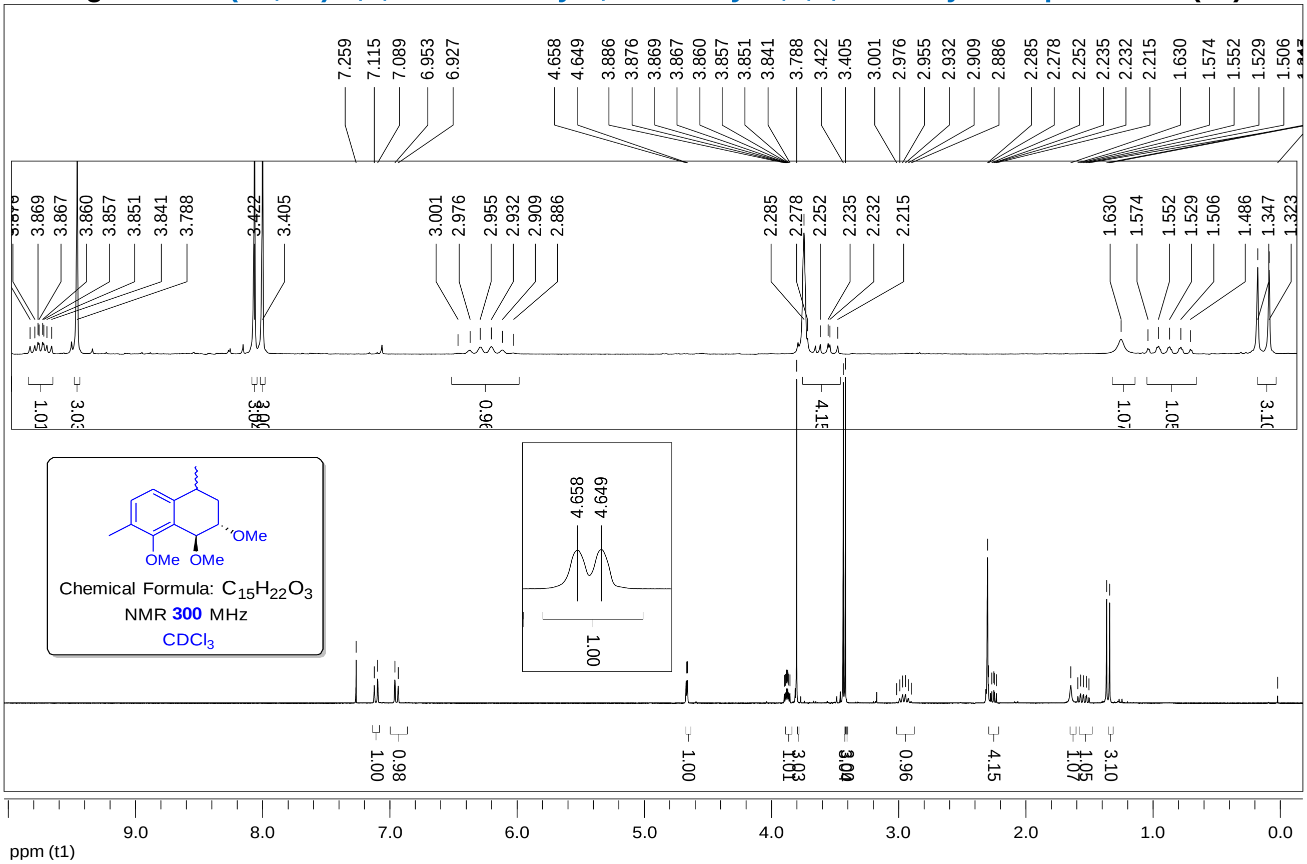

Figure S22: ***(1R,2R)*-1,2,8-Trimethoxy-4,7-dimethyl-1,2,3,4-tetrahydronaphthalene (28).**

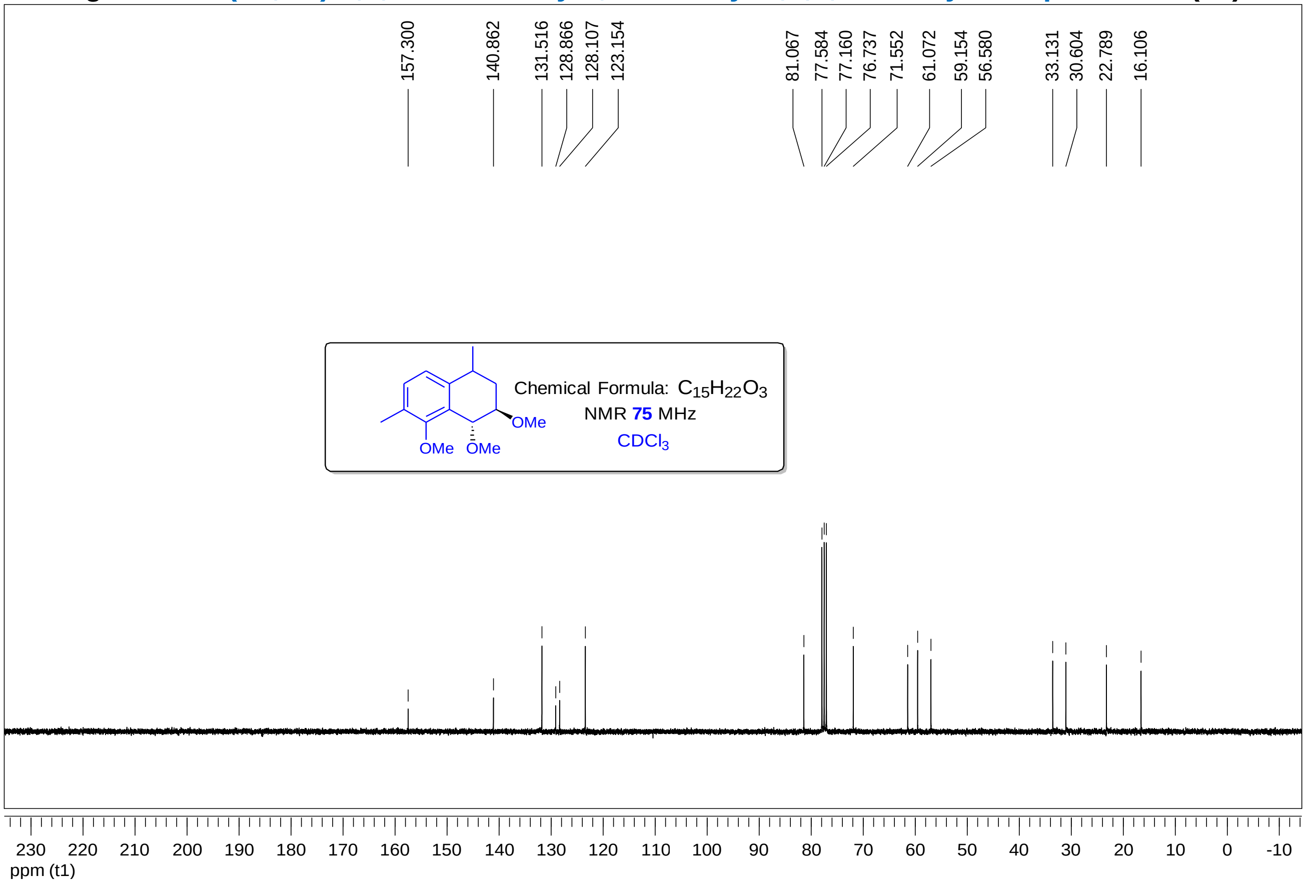

Figure S23: (*1S,2R*)-1,2,8-Trimethoxy-4,7-dimethyl-1,2,3,4-tetrahydronaphthalene (29).

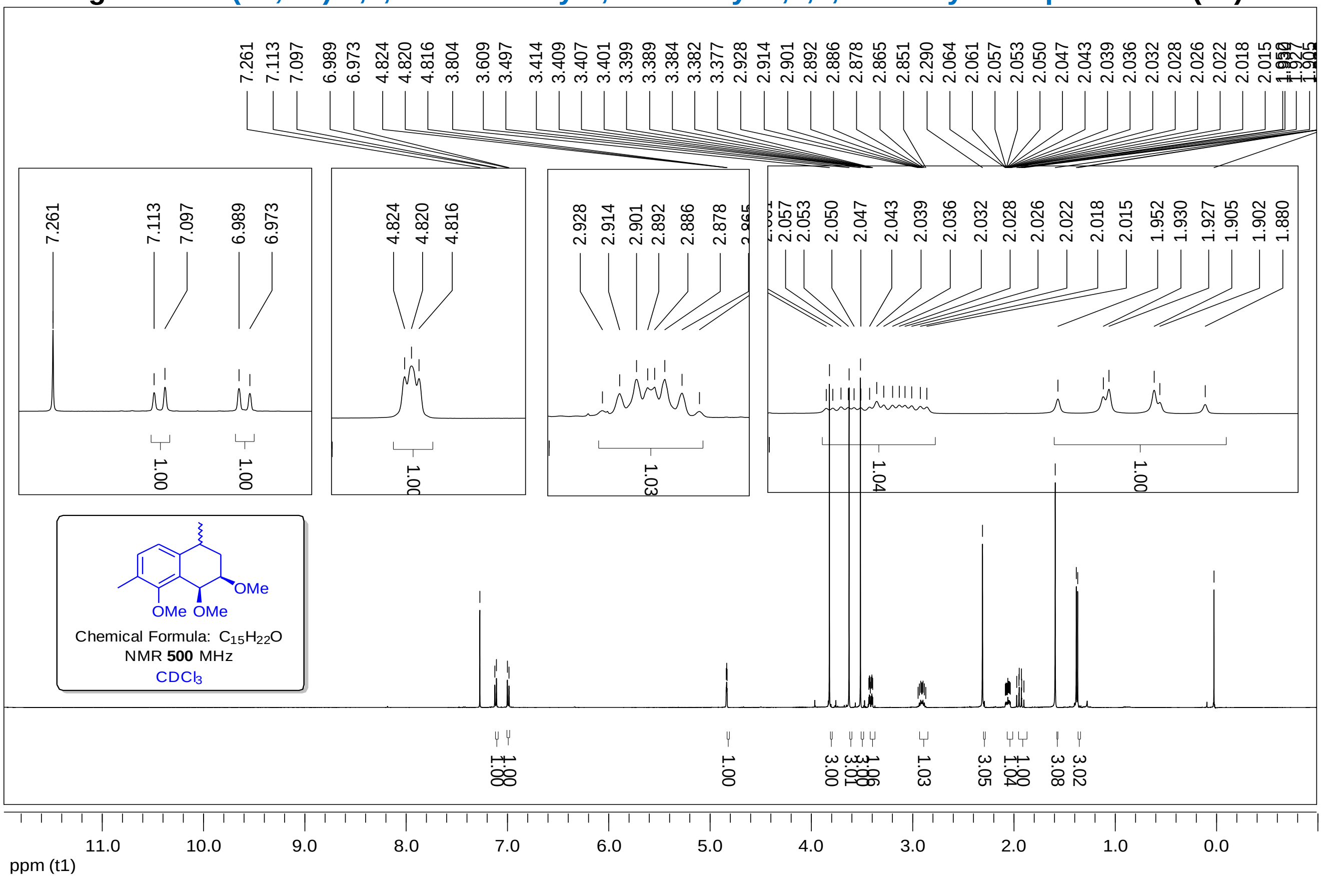

Figure S24: (*1S,2R*)-1,2,8-Trimethoxy-4,7-dimethyl-1,2,3,4-tetrahydronaphthalene (29).

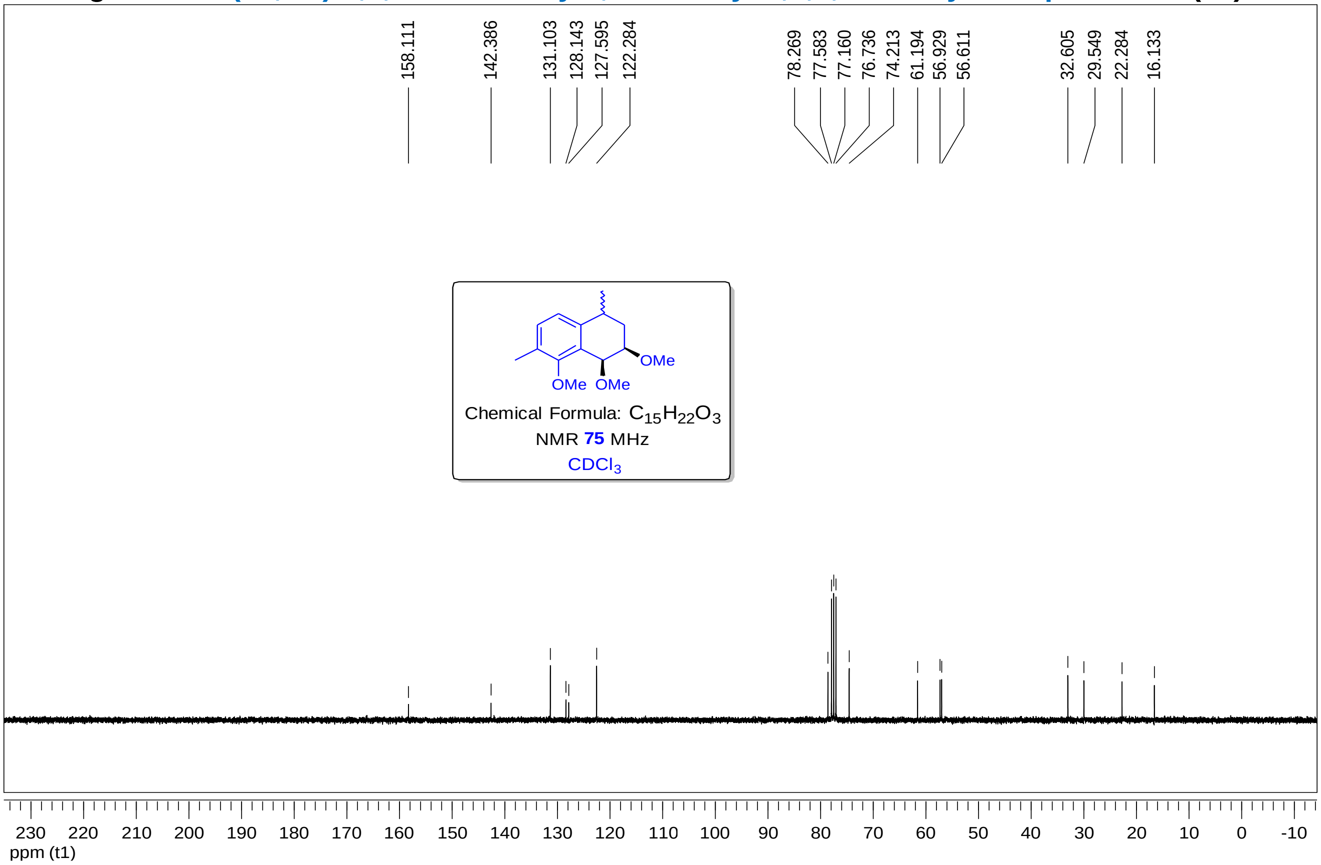

Figure S25: (*3R*)-7-Methoxy-3,6-dimethyl-2,3-dihydro-1H-indene-1-carbaldehyde (**30**).

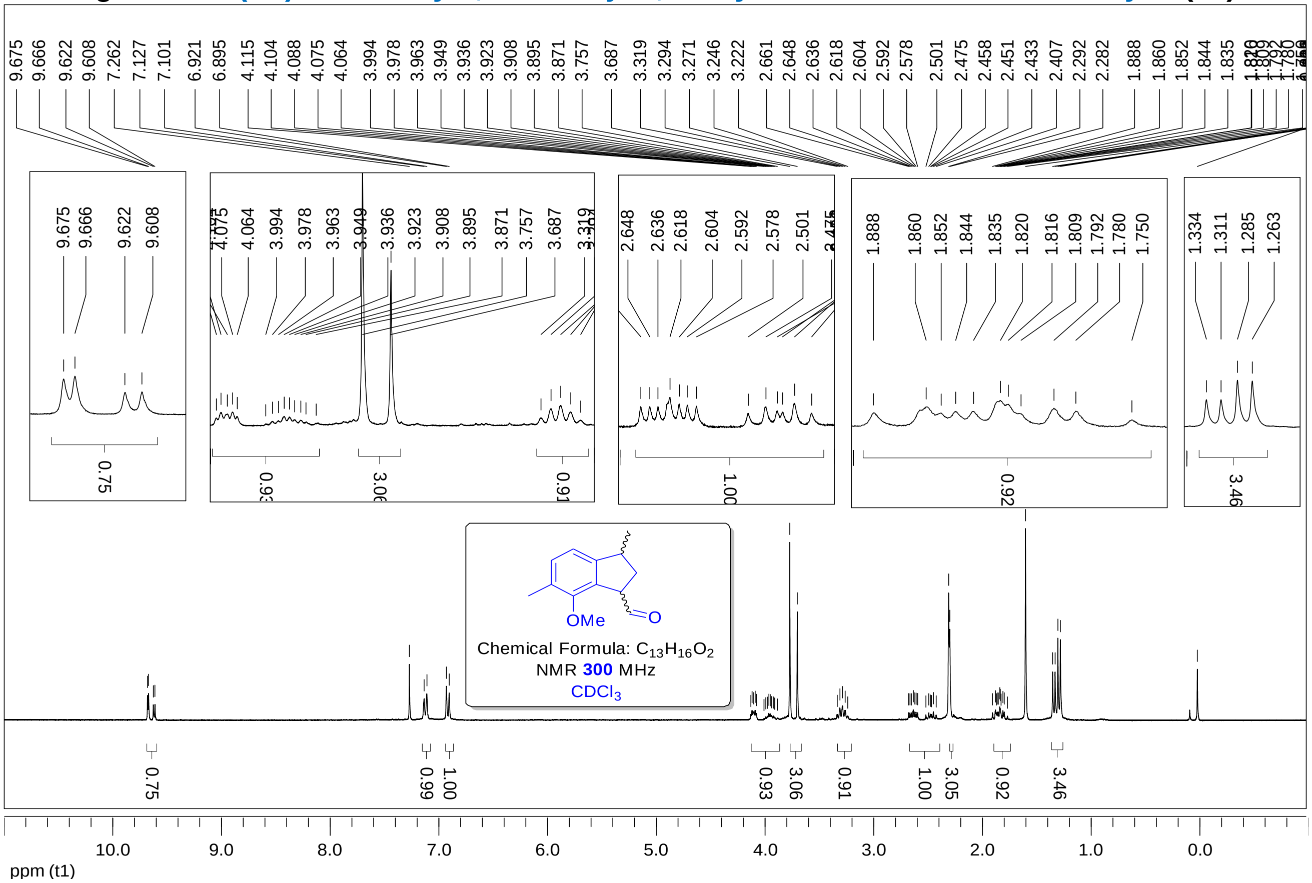

Figure S26: (*3R*)-7-Methoxy-3,6-dimethyl-2,3-dihydro-1H-indene-1-carbaldehyde (**30**).

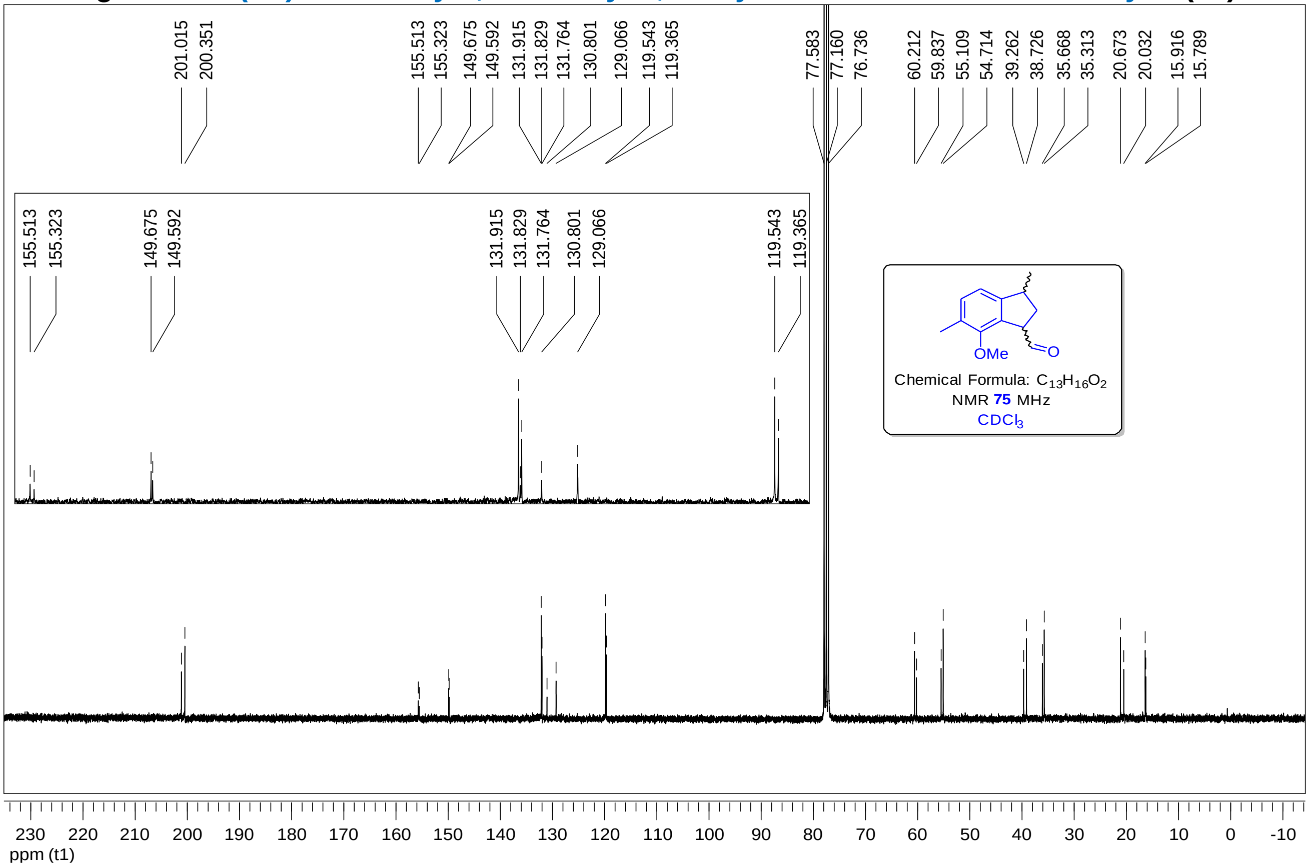

Supplement: Supplementary file 1 [file ao5c03127_si_001.pdf]
